# Analysis of nursing consultations for people with systemic arterial hypertension in Primary Health Care^1^

**DOI:** 10.1590/1518-8345.7998.4876

**Published:** 2026-07-24

**Authors:** Luciana Assis Couto, Carin Caroline Dzembatyi, Dafne Louize Gomes Fernandes, Daiana Bonfim, Andressa Teoli Nunciaroni, Andrea Liliana Vesga Varela

**Affiliations:** 1 Hospital Israelita Albert Einstein, São Paulo, SP, Brazil.; 2 Centro Universitário Campo Real Irati, Irati, PR, Brazil.; 3 Sociedade Beneficente Israelita Brasileira Albert Einstein, São Paulo, SP, Brazil.; 4 Universidade Federal do Estado do Rio de Janeiro, Departamento de Enfermagem de Saúde Pública, Rio de Janeiro, RJ, Brazil.; 5 Scholarship holder at the Fundação de Amparo à Pesquisa do Estado do Rio de Janeiro (FAPERJ), Brazil.

**Keywords:** Hypertension, Primary Health Care, Office Nursing, Nursing Process, Clinical Protocols, Nursing Care

## Abstract

**Objective:**

to analyze nursing consultations with people with systemic arterial hypertension in primary health care according to the Brazilian Guidelines for Arterial Hypertension, the stages of the Nursing Process, and clinical communication.

**Method:**

a cross-sectional analytical study that evaluated 29 nursing consultations performed by 13 nurses with users with arterial hypertension in four Brazilian states. The consultations were assessed using a structured checklist. The data were subjected to descriptive and bivariate analyses, using tables and graphs to characterize the sample and explore potential associations among variables.

**Results:**

there was low adherence to the Nursing Process and the recommendations of the Brazilian Guidelines for Hypertension. Only 20% of the steps in the Nursing Process were fully complied with. Specifically, regarding the Guidelines, there was a lack of fundamental actions, such as blood pressure measurement, risk factor assessment, and communication of the diagnosis.

**Conclusion:**

there was a weakness in the application of the Nursing Process in consultations with people with hypertension. Factors such as case complexity, interruptions, high work demands, gaps in training, and the absence of structured protocols contributed to these findings.

## Highlights

**(1)** Social, cultural, and regional aspects must be part of the nursing process. **(2)** All stages of the Nursing Process and effective communication were covered. **(3)** Hypertension Guidelines can guide the Nursing Process in Primary Health Care. **(4)** The nursing process for people with hypertension needs to be improved in Primary Health Care.

## Introduction

A strengthened Primary Health Care (PHC) system that is adequately sized, territorially based, and involves community participation is associated with health systems that deliver better outcomes and greater access to services for the population^1^ ^-^ ^2^. In Brazil, systemic arterial hypertension (SAH) is one of the most influential factors in premature mortality from cardiovascular diseases (CVD) when associated with dietary, metabolic, and environmental factors^3^. Thus, PHC is a fertile field for implementing evidence-based public policies and early interventions to reduce the incidence and complications of SAH.

In the longitudinal follow-up of patients with SAH in PHC, the Brazilian Guidelines on Hypertension (*Diretrizes Brasileiras de Hipertensão Arterial*) recommend that care include a clinical approach that addresses the psychosocial, cultural, and socioeconomic factors that determine health^4^ ^-^ ^6^. Therefore, the importance of managing SAH in PHC is noteworthy, since this aspect of the Health Care Network considers the conditions that can determine the cardiovascular health and outcomes of the population. 

Thus, in the context of PHC, nurses play a prominent role in care management, particularly in addressing behavioral changes and improving quality of life. In this scenario, studies demonstrate that nursing interventions in the care of people with SAH in PHC are associated with reduced blood pressure levels, improved anthropometric data, treatment adherence, and positive behavioral changes^7^ ^-^ ^9^.

Among the tools used by nurses in PHC, the Nursing Process (NP) stands out. This is a methodological instrument through which the Nursing Consultation^10^ is carried out, contributing to the systematization of clinical reasoning, the identification of health needs and care planning, thereby enabling a care plan that strengthens care coordination, longitudinality, and the evaluation of health outcomes.

Thus, nurses play an essential role in providing user-centered care through interventions that address health needs, thereby fostering a bond of trust^11^ ^-^ ^12^. Still from this perspective, an emerging review of personalized communication strategies points to a positive relationship between customized nursing care and satisfaction/adherence to the agreed treatment^13^, as well as a favorable contribution to building bonds and empowering users in self-care^14^.

Although the NP is legally defined and recognized as the method for implementing nursing care, several challenges remain to its effective use in Brazilian PHC, both related to its operationalization and to the specificities of caring for people with SAH. Thus, to support improvements in clinical practice and consolidate the implementation of the NP in the care of users with SAH in PHC, we seek to understand how nurses have used the elements recommended by the Brazilian Guidelines for Arterial Hypertension in Nursing Consultations, considering the stages of the NP and clinical communication strategies.

Thus, this study aims to analyze nursing consultations for patients with systemic arterial hypertension (SAH) in primary health care (PHC) in accordance with the Brazilian Guidelines for Arterial Hypertension, the stages of the nursing process (NP), and clinical communication.

## Method

The writing of this study followed the Strengthening the Reporting of Observational Studies in Epidemiology (STROBE) checklist^15^.

### Study design and location

This is a cross-sectional analytical observational study based on secondary data from a multicenter study entitled “Advanced practice skills in care management in nursing consultations in Primary Health Care: situational diagnosis and training proposal”, which evaluates different lines of care for nurses in PHC through the recording of 203 nursing consultations performed by 32 nurses. Data collection took place in 17 Basic Health Units (BHUs) in four different federal units, with 6 located in the North, 7 in the Northeast, and 4 in the Southeast of the country.

This study included nursing consultations with individuals who self-reported having SAH and who were recorded in full (n=29), conducted by 13 nurses.

### Population and sample definition

**Figure 1:**
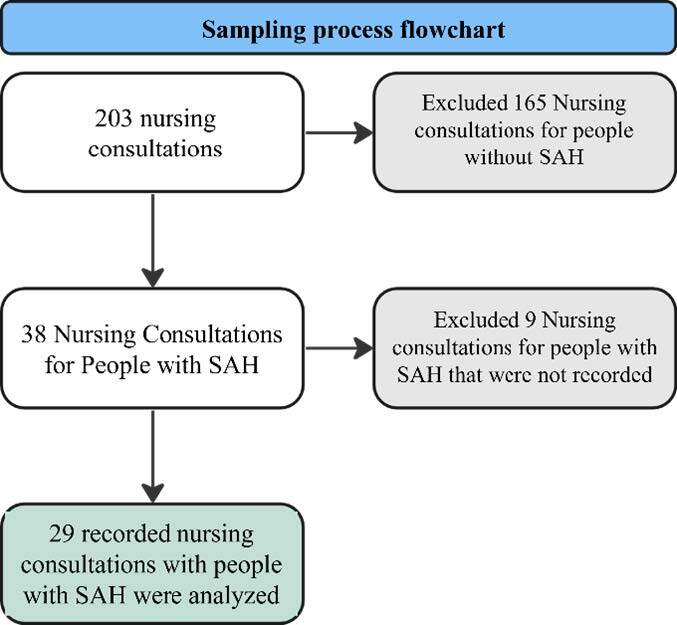
Flowchart showing the sampling process

The sampling process was defined for convenience, considering the inclusion of different scenarios in Brazil (Figure 1). Thus, given the country’s continental size, it was decided to consider three of the five macro-regions in cities with different demographic profiles, morbidity and mortality rates, and service offerings: two large towns and two small cities.

### Selection criteria

The participants in this study were nurses and users present at the recorded nursing consultation. The inclusion criteria for nurses were to have been working in direct care for PHC users for at least one year. For users, the inclusion criterion was that they sought care from a nurse who had agreed to participate in the study.

The nurses and users who agreed to participate in the study signed the Free and Informed Consent Form, the Image and Voice Authorization Form, and the Demographic Characterization Instrument.

### Data collection

Data collection for the multicenter study took place between May and July 2022, following invitations and agreements with management and participating nurses. Two researchers collected the data. The first researcher was responsible for inviting users into the waiting room and obtaining their consent. She also characterized users by sociodemographic data, reasons for seeking nursing consultation, and health history, and nurses by sociodemographic data, training, field characteristics, and professional motivation. The second researcher was responsible for operating the two GoPro^®^ Hero9 cameras: one positioned in the office and the other secured to the nurse’s body with an elastic chest strap.

In addition to the recording, consultation data were extracted from physical or electronic medical records after the consultation. The data were obtained using an instrument developed by researchers, designed to evaluate the recording of the nursing process in medical records. This process enabled evaluation of the stages of the nursing process and clinical reasoning that were not verbally communicated to patients.

Additionally, in this study, data were extracted from recordings of nursing consultations with patients with SAH from September to October 2023, focusing on the evaluation of recommendations from the Brazilian Guidelines for SAH, based on the NP stages.

### Study variables and data collection instruments

A checklist was developed to evaluate nursing consultations regarding care for users with hypertension. This document was based on the Brazilian Guidelines for Hypertension^4^ to support the evaluation of clinical parameters; COFEN (Federal Nursing Council) Resolution 736/2024^10^ and the Nursing Process in Primary Health Care Manual^16^ to evaluate compliance with the stages of the Nursing Process and the communication skills guide Calgary-Cambridge Guide to the Medical Interview - Communication Process^17^ to evaluate the clinical communication strategies used.

The process of constructing the checklist items and sub-items was conducted through joint evaluation by the article’s authors, a team composed of nurses and nutritionists with extensive experience in research and in caring for users who present chronic conditions in PHC. The discussion of the concepts and the integration of the questions that make up the checklist were carried out in meetings attended by at least three researchers. After reaching consensus, a pilot analysis of two nursing consultations was conducted to calibrate the instrument and standardize the evaluators in terms of concepts and responses.

Based on the pilot results, discrepancies among the evaluators’ responses were discussed to reach a final consensus. In these meetings, in addition to analyzing the content, questions were removed, new ones added, and others rewarded as needed. This process resulted in weekly 1h30 sessions for three weeks. After agreement was reached, the process of evaluating all nursing consultations by two nurses (LC and CD) began.

The items and sub-items of the checklist were organized into the following sections: “Nursing Assessment”, “Nursing Diagnosis”, “Nursing Planning”, “Implementation of Nursing Care”, “Nursing Progress”, “Communication”, and “Characterization of the Consultation”. For the analysis, the characterization of the consultation was not considered as a stage of the NP and communication compliance index. The sub-items are questions dependent on the items, for example: for the question “Did the professional perform the correct blood pressure measurement technique?” to be applicable, the answer to the question “Did the professional measure blood pressure?” had to be “yes”. Similarly, for the question “Did the professional request a fasting blood glucose test?” to be applicable, the answer to the question “Did the professional request basic complementary tests?” had to be “yes”.

Each item and subitem was evaluated as present or absent according to the nurse’s actions during the consultation. Additionally, columns for scene evidence and observations were included and are to be completed if necessary. In the “Scene Evidence” column, the corresponding part of the video in which the response was evident was inserted, as well as the minute in which it appeared. The “Observations” field was left open for the evaluator to write any observations they deemed relevant freely. Subsequently, the official video analysis process began, which took about two months to complete in its entirety.

Both the construction of the instrument for evaluating medical records and the checklist for analyzing recorded nursing consultations, as well as data management, were conducted electronically using the RedCap (Research Electronic Data Capture) platform.

To reduce selection bias, inclusion and exclusion criteria were defined. The instrument for analyzing nursing consultations was constructed based on national references in the field of care for people with SAH in PHC, reducing possible measurement biases, as well as the uniform training of researchers who analyzed the videos of nursing consultations. In addition, transparency in the description of procedures and the bivariate analyses employed help to assess the robustness of the results. Together, these measures strengthen the methodological quality and credibility of this study’s conclusions.

### Data processing and analysis

Initially, a univariate descriptive analysis of all variables was performed, using measures of central tendency (mean or median) and dispersion (standard deviation or interquartile range), as appropriate, along with frequency distributions for categorical variables. The normality of the distribution of each continuous variable was formally assessed using the Shapiro-Wilk test. For the bivariate analysis aimed at exploring the relationship between the percentage compliance with the NP steps and the duration of the consultation, a graphical approach complemented by inferential statistics was used. Specifically, a scatter plot was constructed with three elements:

1. points representing each observation,
2. dashed linear regression lines to visualize the trend of the relationship in each subgroup and
3. color distinction based on the categorical variable “Number of Reported Diagnoses”. Considering that the variable “consultation duration” violated the assumption of normality (Shapiro-Wilk, p < 0.05), the strength and direction of the continuous association between these two variables were quantified using the nonparametric Spearman’s rank correlation coefficient (ρ) method.

To assess adherence to the Brazilian Guidelines for Hypertension, a cross-sectional observational analysis was conducted, using the PE as the methodological reference framework. A data extraction checklist was constructed by aligning each stage of the process-Assessment, Diagnosis, Planning, Implementation, and Nursing Evolution-with the specific recommendations of the Guidelines. Through direct observation of 29 consultations, the execution of critical items for the management of SAH was verified, such as the correct technique for measuring blood pressure, the investigation of cardiovascular risk factors, the performance of a targeted physical examination, health education, and the establishment of a shared care plan. Thus, the NP served as an analytical lens to systematically and comprehensively measure whether the practices observed corresponded to the recommended standard of clinical excellence.

The percentage of compliance with the PE stages was calculated as the number of items analyzed, assuming equal weights across all stages. To this end, the total number of items that entirely comprise the NP was determined, ensuring an equitable distribution across the stages. These percentages were described and graphically presented according to user and nursing professional characteristics and consultation characteristics. All statistical analyses were performed using statistical software R version 4.1.3 (R Project for Statistical Computing) together with statistical software R Studio version 1.4.1717 (RStudio).

### Ethical considerations

Ethical and legal requirements were met, in accordance with Resolution No. 466/2012 of the National Health Council. The study was approved by the Research Ethics Committee (56255622.2.0000.0071) and received consent from the participating centers. In accordance with Open Science, the data are available upon consultation with the authors, in part, since the recording of nursing consultations can identify the nurses, users, and participating health units.

## Results

### Users

This study included 29 recordings of nursing consultations concerning users of Primary Health Care units who self-reported a diagnosis of SAH. The users included in the analyses had a mean age of 56.62 years (±15.28), with 86.21% female and 68.96% Black or Brown.

In terms of socioeconomic conditions, 72.41% of users received some income, and of these, 75.87% received between 0 and 2 minimum wages. Regarding their places of origin, three users were from the municipality located in the state of Alagoas (AL), 11 from the state of Amazonas (AM), six from Rio Grande do Norte (RN), and nine from the state of São Paulo (SP). In addition, 82.76% reported living with family members. In addition to hypertension, 55.27% of users reported having more than three different diagnoses. The main reasons for seeking health services were acute events (30.37%), cervical cytopathological testing (17.24%), and evaluation of test results (10.34%).

### Nurses

A total of 13 nurses were responsible for consultations with users with SAH, including one from AL, four from AM, three from RN, and five from SP. Of these, 76.92% were female. The health services in which they worked were, in 61.54% of cases, Basic Health Units under the Family Health Strategy, and among the 13 professionals participating in the study, 8 (61.5%) had at least 5 years of experience in the field. All professionals reported holding a postgraduate degree; among these, 91.54% had residency or specialization in PHC, Family and Community Health, Collective Health, or Public Health.

Concerning factors that facilitated or hindered the consultations, 76.92% of nurses had an exclusive room and 61.54% had access to some taxonomy as a guide for nursing diagnoses. Further information about the characteristics of the nurses whose consultations were evaluated in this study is shown in Table 1. As further illustrated in this table, there were cases where nurses did not report, or reported participating in more than one course in the last year and applying multiple protocols when providing care to users.

**Table 1:** Professional profile of practice and training of nurses working in health services (n = 13). Brazil, 2022-2023

Variables	% (n)*
**Salary** 1 to 2 MW^†^ 3 to 4 MW^†^ 5 to 6 MW^†^ > 7 MW^†^	15.38 (2) 23.08 (3) - 61.54 (8)
**Another employment relationship** Yes No	38.46 (5) 61.54 (8)
**Type of team at the Unit** Family Health Strategy BHU^‡^ Mixed BHU^‡^ Traditional BHU^‡‡^ Rural	61.54 (8) 15.38 (2) 15.38 (2) 7.69 (1)
**There is a Multidisciplinary Team at the Unit** Yes No	61.54 (8) 38.46 (5)
**Length of experience as a nurse at PHC** ^§^ Between 1 and 5 years Between 6 and 10 years > 10 years	3.46 (5) 46.15 (6) 15.38 (2)
**There are difficulties in conducting consultations.** Yes No	69.23 (9) 30.77 (4)
**Standardized instruments for the nursing process** Yes No	38.46 (5) 61.54 (8)
**Taxonomy used for Nursing Diagnosis** NANDA^||^ CIPE^¶^ Others None	23.08 (3) 30.77 (4) 7.69 (1) 38.46 (5)
**Professional motivation** With satisfaction Without satisfaction	76.92 (10) 23.08 (3)
**Residency or Specialization in PHC** ^§^ Yes No	30.77 (4) 69.23 (9)
**Specialization in Family and Community Health, Collective Health, or Public Health** Yes No	61.54 (8) 38.46 (5)
**Number of Professionals with master’s Degrees** Yes No	30.77 (4) 69.23 (9)
**Participation in courses in the last year** Health of the Elderly Care for chronic patients (SAH**/DM^††^) Nursing Process	23.08 (3) 30.77 (4) 30.77 (4)
**Took classes on the Nursing Process** Yes No I do not know	84.62 (11) 7.69 (1) 7.69 (1)
**In your unit, do you use Nursing Protocols?** Yes No	46.15 (6) 53.85 (7)
**Protocols used in Elderly Health Care** Ministry of Health Primary Care Handbook State Protocol Protocol of other municipalities COREN Protocol^‡‡^ Protocol of your municipal health department	76.92 (10) 7.69 (1) 7.69 (1) - 15.38 (2)
**Protocols used for chronic care** Ministry of Health Primary Care Handbook Ministry of Health Protocol State Protocol Protocol of other municipalities COREN Protocol^‡‡^ Protocol of your Municipal Health Department	76.92 (10) 61.54 (8) 23.08 (3) 7.69 (1) - 15.38 (2)

*%(n) = Percentage value (gross number); ^†^MW = Minimum wage, corresponding to R$1,518.00 in Brazil, year 2025; ^‡^BHU = Basic Health Unit; ^§^PHC = Primary Health Care; ^||^NANDA = North American Nursing Diagnosis Association; ^*¶*^ ICNP *=* International Classification for Nursing Practice; **SHA = Systemic Arterial Hypertension; ^††^DM = Diabetes Mellitus; ^‡‡^COREN = Regional Nursing Council

The average duration of each consultation was 22.89 minutes (±13.54), 66.67% of consultations were interrupted, 55.17% were scheduled in advance, and 24.14% of consultations involved a user complaint related to SAH.

The following description is based on the content generated by analyzing the consultation videos using the checklist. In the Nursing Assessment stage, 72.41% of professionals did not measure blood pressure during the consultation and, among those who did, 25% performed the procedure correctly. The search for anthropometric data was absent in 51.72% of consultations, as was cardiac auscultation (86.21%). Assessment of users’ extremities was documented in 10% of consultations, in which professionals assessed the possibility of edema.

The professionals performed basic complementary tests in 31.0% of consultations, and in 88.89% of these cases, the results were communicated to the user. The use of continuous medication was investigated in 51.72% of consultations, and in none of these cases was the use of non-prescription medication and/or illicit drugs questioned.

Specific risk factors for actual cardiovascular disease were explored in 72.42% of consultations, with age being the most frequent (66.67%). The presence of smoking, alcohol consumption, or sodium intake was not explored in 94.24%, 95.25%, and 95.24% of consultations, respectively. Coronary artery disease and chronic kidney disease were present in 1 and 3 consultations, respectively. The investigation of the presence of diabetes was evident in 42.86% of consultations.

In 79.31% of cases, psychosocial, cultural, and socioeconomic aspects that could be associated with blood pressure measurements and comorbidities were not investigated, and of those that did include such an investigation, emotional stress was the most frequent (66.67%).

The following were absent in 100% of consultations: evaluation of Ankle-Brachial Index (ABI), auscultation of carotid arteries, calculation of Glomerular Filtration Rate (GFR), neurological examination, history of stroke (or Cerebrovascular Accident) and investigation of hypertensive retinopathy, calculation of Pulse Wave Velocity (PWV), assessment of the apical impulse on palpation, investigation of family history of early onset of SAH, investigation of sleep apnea and pulse pressure.

The communication of the Nursing Diagnosis was not evident in any of the consultations. During the Planning stage, 89.66% of professionals did not set goals, and 20.69% of consultations involved joint planning with the user.

Nursing prescriptions were not verbalized in 75.86% of consultations, and in none of them were such prescriptions delivered in writing to the user. Finally, the calculation of cardiovascular risk was not evident in any of the consultations. Sixty-five point sixty-two percent of the consultations were follow-up consultations, and the Nursing Evolution stage was not explicit in any of them.

Regarding the communication and interpersonal skills of the professionals, 65.52% identified the user and 86.21% did not introduce themselves. Comfort and privacy were guaranteed in 96.55% of consultations, and interviews began with open-ended questions in 51.72%. In addition, 79.31% of professionals maintained eye contact with the user during the interaction and gave the user sufficient time to express themselves without interruption.

In addition, 58.62% of professionals practiced active listening, and 93.1% used language adapted to the educational and socioeconomic conditions of users. Verification of the person’s understanding and encouragement to clarify doubts were present in 79.31% of consultations, and the summarization technique was applied in 55.17% of clinical encounters. Furthermore, 75.86% of users were not involved in planning therapeutic options regarding their own health-illness-care process, and user feedback on the care provided was not requested in 89.66% of consultations.

### Nursing Process

*According to the execution*

Table 2 shows that, in summary, of the total of 48 items evaluated by the checklist, the median number of items fulfilled was 18, indicating that 50% of nurses fulfilled up to 18 items of the Nursing Process. This value corresponds to a median fulfillment rate of 20% across the total number of steps in the NP.

**Table 2:** Distribution of compliance with the stages of the Nursing Process, according to items proposed by the Brazilian Guidelines for Arterial Hypertension (N = 29). Brazil, 2022-2023

Variables	Yes % (n)*	No % (n)*
**Nursing assessment performed by professionals**		
Did you check your blood pressure during the appointment?	27.59 (8)	72.41 (21)
Did you look up the user’s anthropometric data?	48.28 (14)	51.72 (15)
Did you perform auscultation of the carotid arteries?	-	100.0 (29)
Did you perform cardiac auscultation?	13.79 (4)	86.21 (25)
Did you count your heart rate?	3.45 (1)	96.55 (28)
Did you examine the extremities?	10.34 (3)	89.66 (26)
Did you perform an ABI assessment?	-	100.0 (29)
Checked results of basic complementary tests related to hypertension or CVR^‡^?	31.03 (9)	68.97 (20)
Did you assess renal function by calculating GFR^§^ using CKD-EPI^||^ or MDRD^¶^?	-	100.0 (29)
Examined abdomen?	6.9 (2)	93.1 (27)
Did you perform a neurological examination?	-	100.0 (29)
Have you researched medications that are used continuously?	51.72 (15)	48.28 (14)
Have you investigated the use of medications without a prescription?	-	100.0 (29)
Did you investigate illicit drug use?	-	100.0 (29)
Did you investigate specific risk factors for actual cardiovascular disease?	72.41 (21)	27.59 (8)
Have you investigated factors that may modify the risk for hypertensive users?	27.59 (8)	72.41 (21)
Did you investigate psychosocial, cultural, and socioeconomic aspects that may be interfering with the measured values/comorbidities?	20.69 (6)	79.31 (23)
Did you perform a PWV** assessment?	-	100.0 (29)
Assessment of the apical impulse on palpation?	-	100.0 (29)
**Nursing Diagnosis**		
Listed nursing diagnosis(es):	-	100.0 (29)
Did you communicate the listed diagnosis(es) to the user?	-	100.0 (29)
**Nursing Planning**		
Have you determined the results you hope to achieve and/or set goals?	10.34 (3)	89.66 (26)
Have you established nursing actions or interventions that will be carried out to achieve the goals?	33.33 (1)	66.67 (2)
**Implementation of Nursing Care**		
Does the user need to request basic complementary tests?	17.24 (5)	82.76 (24)
Did you issue a nursing prescription? (Whether written or verbal)	24.14 (7)	75.86 (22)
Of those who issued a prescription (n=7), was it given to the user in written form on paper?	-	100.0 (7)
**Evolution of Nursing**		
Did you evaluate nursing care?	-	100.0 (29)
Have you listed new nursing diagnoses for the presenting need?	-	100.0 (29)

*%(n) = Percentage value (gross number); ^†^ABI = Ankle-Brachial Index; ^‡^CVR = Cardiovascular Risk; ^§^GFR = Glomerular Filtration Rate; ^||^CKD-EPI = Chronic Kidney Disease Epidemiology Collaboration, equation for estimating glomerular filtration rate; ^¶^MDRD = Modification of Diet in Renal Disease, formula for estimating glomerular filtration rate; **PWV = Pulse Wave Velocity

**Figure 2:**
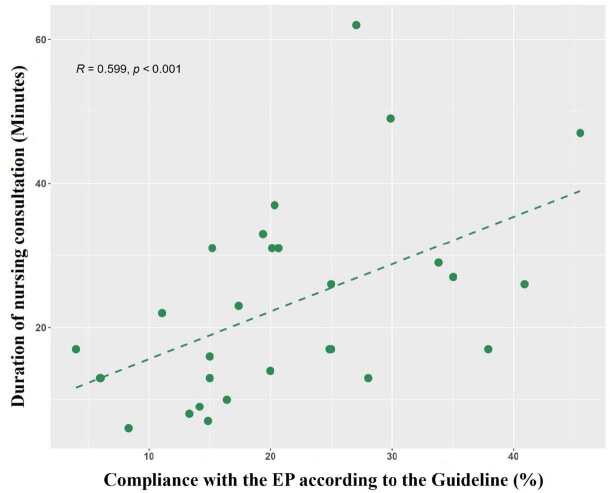
Correlation between the duration of nursing consultations and compliance with the stages of the Nursing Process for users with Arterial Hypertension in Primary Health Care. Brazil, 2022-2023

As illustrated in Figure 2, a positive and statistically significant correlation was observed between longer nursing consultation times and greater adherence to the stages of the Nursing Process (NP) in patients with SAH. Analysis of consultation time using the Shapiro-Wilk test confirmed the absence of normality in its distribution (p = 0.0121). Given this result, we opted to use Spearman’s nonparametric test to assess the correlation.

**Figure 3:**
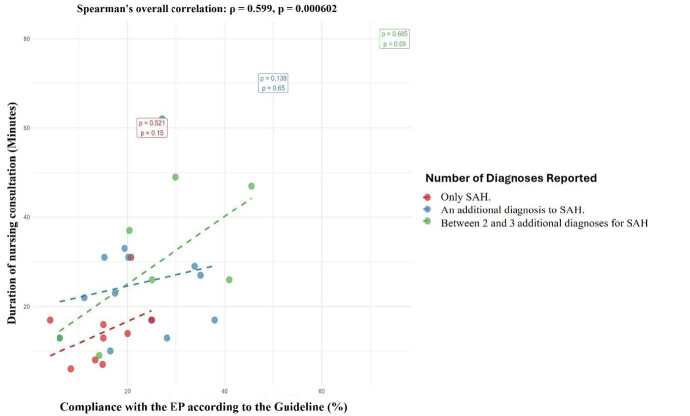
Correlation between diagnoses associated with high blood pressure and the consultation time required to complete the stages of the nursing process. Brazil, 2022-2023

The analysis stratified by diagnostic complexity (Figure 3) revealed a gradient in correlation magnitude, although none of the subgroups were statistically significant (p > 0.05). Specifically, a moderate positive correlation (ρ = 0.521; p = 0.150) was observed in the group with only SAH, a feeble positive correlation (ρ = 0.138; p = 0.653) in the group with an additional diagnosis, and a strong positive correlation (ρ = 0.685; p = 0.090) in the group with greater complexity (2-3 additional diagnoses). This ascending pattern suggests that the relationship between consultation time and adherence to the NP intensifies progressively with increasing clinical complexity.

*According to the record*

According to the NP stages, 79.31% of consultations were documented in medical records, and the content was legible in 87.5% of the records. In the Assessment, subjective data were absent in 52.17% of consultations, and objective data were absent in 86.96% of them. 73.91% of consultations lacked a nursing diagnosis record, and 95.65% of these consultations included at least one diagnosis according to the International Classification of Primary Care (ICPC).

Regarding the Planning stage, in 95.65% of consultations there was no record of goals, and of those that did, one was actually related to the nursing diagnosis listed above. Nursing prescriptions were documented in two consultation records, which were consistent with the previously established nursing goals and diagnoses. The Implementation stage was absent in 95.65% of records, and the Nursing Evolution stage was evident in 50% of them.

## Discussion

The results of this study indicate a significant gap between the practice observed in Primary Health Care and the standards of care recommended by the Brazilian Guidelines for Hypertension, when analyzed through the lens of the Nursing Process (NP). The low frequency of essential assessment components-such as technical blood pressure measurement, investigation of modifiable risk factors (such as sodium intake and physical inactivity), and targeted physical examination-suggests that care is being provided in a mechanized and fragmented manner, failing to take advantage of the consultation as an opportunity to obtain a complete and accurate picture of the user’s cardiovascular risk profile.

The almost complete absence of nursing diagnoses (100% did not list any) is particularly concerning, as it indicates that clinical judgment is not being formalized, thereby preventing care planning from being specific, individualized, and grounded in the identified health needs. This weakness was revealed from the moment of the Nursing Assessment, which was carried out in an incomplete manner. Consequently, considering that the stages of the NP are interrelated and interdependent, the implementation of subsequent stages is compromised. This result is consistent with the findings of a study that conducted a situational diagnosis of the performance of nurses in PHC in Santa Catarina^18^ in 2023, which concluded that the application of the NP in nursing consultations still occurs in incipient and fragmented manner.

The disconnect between data collection and subsequent stages of the NP results in an incomplete care cycle. The absence of systematic clinical diagnoses compromises planning, which is often nonspecific or absent, as evidenced by the low rate of joint goal setting (10.34%). This scenario has already been described in other contexts, in which the development of individualized care plans is a differential in care practice, thereby favoring adherence and control of hypertension^19^ ^-^ ^20^. Consequently, the nursing interventions implemented lack a theoretical basis and clear direction, focusing on requests for tests and generic prescriptions, to the detriment of educational and behavioral strategies. However, robust evidence indicates that educational interventions led by nurses significantly reduce blood pressure levels and promote self-care^8^ ^,^ ^21^ ^-^ ^22^.

The high proportion of professionals who did not provide feedback or verify the user’s understanding (89.66%) reinforces a one-way communication, which does not promote shared responsibility. Qualitative studies also show that, in practice, nursing consultations tend to be uncreative and professional-centered, limiting users’ active participation^23^. Thus, it is observed that the gap in the integration between diagnosis, planning, and intervention directly affects the effectiveness of care in chronic conditions, reinforcing the need to reorganize the workflow to ensure user-centered and evidence-based care.

To analyze the factors related to the application of the NP in its entirety, the characteristics of the users served, the nurses, and the consultations evaluated were considered. An important aspect was the consultation time, as it became evident that people with multiple comorbidities need a longer consultation time so that all their health needs can be addressed. In addition, there were interruptions in most consultations, which disrupted clinical reasoning and impaired time management, as evidenced by an integrative literature review conducted in 2020^24^.

This, in turn, leads to the context of clinical communication, an essential component of nursing consultations, but one assessed in this study as still somewhat secondary. Actions such as communicating diagnoses and therapeutic goals, providing written nursing prescriptions, and evaluating care together with the user are essential for effective clinical communication^25^ ^-^ ^26^, treatment adherence^27^, and improved bonding and satisfaction^28^. However, managing the schedule and addressing external interference, which ultimately falls under the responsibility of nurses in PHC, remains a challenge for the full implementation of the NP and for the execution of nursing consultations that include all items recommended by the SAH Guidelines^29^. 

An analysis of nursing consultations carried out in Rio de Janeiro in 2021 showed that professionals were overwhelmed by the pressure of care due to busy schedules, which resulted in a medical-centered care model focused mainly on complaints and behavior^30^, making it difficult to use the NP in its entirety and potentially interfering negatively with users’ clinical outcomes.

Notably, there is a failure to comply with the Nursing Diagnosis stage. In our study, this stage was present in 26.09% of the consultations evaluated, and this fact is justified by the lack of skill and management with classificatory taxonomies. However, 38.5% of the evaluated professionals reported not using any taxonomy for nursing diagnosis. Such evidence is consistent with results in other contexts, where the difficulty of implementing the diagnostic stage in the NP remains strongly marked by theoretical complexity, gaps in training, lack of institutional support, high workload, and low practical mastery^31^ ^-^ ^32^.

Although most professionals report having received training in Nursing Process during their undergraduate studies and having specialization and/or residency, there is a gap between theory and the actual use of this tool by professionals, with the main hindering factor being the lack of training and implementation of knowledge in practice^33^, understanding that the NP is a tool for safe nursing practice. It is therefore necessary to be familiar with the taxonomies that standardize specific nursing care, as well as standards of care in Health Programs, since general protocols, for example, do not guarantee the NP’s full integration into the clinical encounter during consultation.

Thus, although many professionals participating in the study reported using some protocol to guide care, more than half of them were not specific to nursing, which may be an obstacle to the consolidation of a qualified NP^34^. The use of nursing protocols is essential, as they assist in decision-making focused on patient safety and support professionals by considering the ethical principles of the profession^35^. Implementing protocols as strategies to reduce obstacles and strengthen the process^36^ would be of great importance, given that 53.85% of nurses reported not using Nursing Protocols in their care practice.

In large municipalities in the interior of the state of São Paulo^37^, in 2015, the NP in a PHC unit was still in its infancy, with 19% of nurses reporting that they did not use it, 38% reporting that they used it rarely, and 29% reporting that they used it sporadically. Compared with our results, there was an improvement in the use of the NP, even though it does not cover all five stages. Thus, it is essential that, in addition to the development of clinical guidelines and protocols, the time required to complete the planned activities be calculated and evidenced, as this could even imply the degree of recommendation of a given intervention, the prioritization of more serious cases, and the need to increase the number of professionals available to care for these users^38^.

Despite the challenges for NP in primary care when caring for people with SAH, nursing consultations are a care practice that favors longitudinal care, since interventions are based on scientific evidence and address clinical, social, cultural, and regional aspects of care in a personalized manner^39^ ^-^ ^41^.

Starting from the NP, based on theories and used in its entirety, developed together with the person, on an individual basis, there is greater satisfaction with health monitoring, which favors behavioral changes and improves the clinical situation and quality of life^42^ ^-^ ^43^. For professionals, despite the challenges, nursing consultations increase nurses’ autonomy and yield positive experiences in their work^44^ ^-^ ^45^. Within the sphere of public policies and the Unified Health System (SUS, as per its Portuguese acronym), PHC can be further strengthened, with an expressive contribution from nursing toward reducing the burden of non-communicable chronic diseases.

This study has some limitations. First, the sample size was not calculated, and the selection of units was based on convenience. However, units from four different municipalities in the country were included, representing particular sociodemographic characteristics of regions of Brazil. The second limitation concerns the number of consultations analyzed (n=29) and the number of nurses (n=13), resulting in heterogeneous professional characteristics and a limited sample. The possible information bias related to the instrument used to evaluate nursing consultations may be a limitation.

Finally, as in observational studies, there is the possibility of selection bias attributable to nurses who agreed to participate, who may have different practices than those who refused to participate in the study. In addition, there is the possibility of social desirability bias, which may be triggered by behavioral change due to participating in the research and being subject to recording and observation. Despite its limitations, this study discusses essential results regarding the evaluation of all stages of the NP in PHC in the care of people with SAH. The findings contribute to critical reflection on nursing care and underscore the importance of recording the NP. It presents the factors associated with the challenges of using the NP, which will guide actions and research and allow for the adaptation of documents and public policies to address the specificities of nursing in PHC.

Given the still scarce number of studies conducted in Brazil on this topic, this research, as a primary study, paves the way and illuminates future discussions on the production of instruments and knowledge that strengthen clinical nursing practice. The aim is to enhance the rationale for the professional category’s existence: to provide the best care to users, grounded in robust instruments and the best scientific evidence.

## Conclusion

Weaknesses were evident in the assessment of the elements recommended by the Brazilian Guidelines for Hypertension and in the consolidation of the NP during nursing consultations with people with SAH. The complexity of users, time, and interruptions during consultations, high demand for care, professional training, and the lack of structured nursing protocols based on the stages of the NP were hypothesized to explain this finding. In addition, the publication of care guidelines without a document outlining their applicability impedes their implementation in daily practice.

## Data Availability

All data generated or analysed during this study are included in this published article.
