# Supplementary material for: Analysis of nursing consultations for people with systemic arterial hypertension in Primary Health Care
Source: Rev Lat Am Enfermagem. 2026 Jul 24;34:e4876. doi: 10.1590/1518-8345.7998.4876 (PMC13405592; doi:10.1590/1518-8345.7998.4876)
Supplement: Translation

# Translation: Análise da consulta de enfermagem a pessoas com hipertensão arterial sistêmica na Atenção Primária à Saúde^*^

**DOI:** 10.1590/1518-8345.7998.4877

**Authors:** Luciana Assis Couto, Carin Caroline Dzembatyi, Dafne Louize Gomes Fernandes, Daiana Bonfim, Andressa Teoli Nunciaroni, Andrea Liliana Vesga Varela

**Affiliations:** 1 Hospital Israelita Albert Einstein, São Paulo, SP, Brasil.; 2 Centro Universitário Campo Real Irati, Irati, PR, Brasil.; 3 Sociedade Beneficente Israelita Brasileira Albert Einstein, São Paulo, SP, Brasil.; 4 Universidade Federal do Estado do Rio de Janeiro, Departamento de Enfermagem de Saúde Pública, Rio de Janeiro, RJ, Brasil.; 5 Scholarship holder at the Fundação de Amparo à Pesquisa do Estado do Rio de Janeiro (FAPERJ), Brasil.

**Keywords:** Hipertensão Arterial Sistêmica, Atenção Primária à Saúde, Enfermagem de Consultório, Processo de Enfermagem, Protocolos Clínicos, Cuidados de Enfermagem.

## Abstract

**Objetivo:**

analisar a consulta de enfermagem em pessoas com Hipertensão Arterial Sistêmica na Atenção Primária à Saúde de acordo com as Diretrizes Brasileiras de Hipertensão Arterial, as etapas do Processo de Enfermagem e a comunicação clínica.

**Método:**

estudo transversal analítico que avaliou 29 consultas de enfermagem realizadas por 13 enfermeiros aos usuários com hipertensão arterial em quatro estados brasileiros. A avaliação das consultas foi feita por meio de um *checklist* estruturado. Os dados foram submetidos à análise descritiva e bivariada, utilizando tabelas e gráficos para caracterizar a amostra e explorar possíveis associações entre as variáveis.

**Resultados:**

houve baixa adesão ao Processo de Enfermagem e às recomendações das Diretrizes Brasileiras de Hipertensão. Apenas 20% das etapas do Processo de Enfermagem foram integralmente cumpridas. Especificamente em relação às Diretrizes, observou-se a insuficiência de ações fundamentais, como a aferição da pressão arterial, a avaliação de fatores de risco e a comunicação clínica do diagnóstico.

**Conclusão:**

houve fragilidade na aplicação do Processo de Enfermagem nas consultas às pessoas com hipertensão. Fatores como a complexidade dos casos, interrupções, alta demanda de trabalho, lacunas na formação e ausência de protocolos estruturados contribuíram para esses achados.

## Destaques

**(1)** Aspectos sociais, culturais e regionais precisam compor o Processo de Enfermagem. **(2)** Todas as etapas do Processo de Enfermagem e comunicação efetiva foram contempladas. **(3)** As Diretrizes de Hipertensão podem nortear o Processo de Enfermagem na Atenção Primária à Saúde. **(4)** O Processo de Enfermagem à pessoa com hipertensão precisa ser aprimorado na Atenção Primária à Saúde.

## Introdução

Uma Atenção Primária à Saúde (APS) fortalecida, adequadamente dimensionada, com base territorial e com participação comunitária, está associada aos sistemas de saúde com melhores resultados e maior acesso da população aos serviços^1^ ^-^ ^2^. No Brasil, a hipertensão arterial sistêmica (HAS) é um dos fatores mais influentes na mortalidade precoce por doenças cardiovasculares (DCV) quando associada aos fatores dietéticos, metabólicos e ambientais^3^. Desta forma, a APS é campo fértil para implementação de políticas públicas e intervenções precoces baseadas em evidências a fim de diminuir as tendências da incidência e de complicações relacionadas à HAS.

No seguimento longitudinal da pessoa com HAS na APS, é recomendado pelas Diretrizes Brasileiras de Hipertensão Arterial que o cuidado contemple abordagem clínica, aspectos psicossociais, culturais e socioeconômicos que determinam a saúde^4^ ^-^ ^6^. Denota-se, portanto, a importância do manejo da HAS na APS, uma vez que este ponto da Rede de Atenção à Saúde considera as condições que podem determinar a saúde e os desfechos cardiovasculares da população. 

Assim, no contexto da APS, a enfermeira ocupa um papel de destaque na gestão do cuidado, em especial no que concerne às mudanças comportamentais e melhoria da qualidade de vida. Neste cenário, estudos demonstram que as intervenções de enfermagem no cuidado à pessoa com HAS na APS estão associadas à redução de níveis pressóricos, melhora de dados antropométricos, adesão ao tratamento e mudanças comportamentais positivas^7^ ^-^ ^9^.

Entre as ferramentas de trabalho da enfermeira na APS, destaca-se o Processo de Enfermagem (PE). Trata-se de instrumento metodológico por meio do qual se concretiza a Consulta de Enfermagem^10^ e que contribui para a sistematização do raciocínio clínico, identificação das necessidades de saúde e planejamento do cuidado, possibilitando um plano de cuidado que fortaleça a coordenação do cuidado, a longitudinalidade e a avaliação dos resultados de saúde. 

Desta forma, a enfermeira tem papel importante na oferta do cuidado centrado no usuário com intervenções frente às necessidades de saúde, o que possibilita a criação de um vínculo de confiança^11^ ^-^ ^12^. Ainda nesta perspectiva, uma revisão emergente sobre estratégias de comunicação personalizada aponta relação positiva entre cuidados de enfermagem personalizados e satisfação/adesão ao tratamento pactuado^13^, bem como contribuição favorável na construção do vínculo e empoderamento dos usuários no autocuidado^14^. 

Embora o PE seja legalmente determinado e reconhecido como o método para a implementação do cuidado de enfermagem, ainda estão presentes diversos desafios para a sua efetiva utilização na APS brasileira, tanto relacionados à sua operacionalização como em relação às especificidades no cuidado a pessoas com HAS. Assim, a fim de fornecer subsídios para aprimorar a prática clínica e consolidar a implementação do PE nos atendimentos aos usuários com HAS na APS, busca-se compreender como as enfermeiras têm utilizado os elementos preconizados pelas Diretrizes Brasileiras de Hipertensão Arterial nas Consultas de Enfermagem, considerando as etapas do PE e estratégias de comunicação clínica.

Deste modo, o presente estudo tem como objetivo analisar a consulta de enfermagem em pessoas com Hipertensão Arterial Sistêmica (HAS) na Atenção Primária à Saúde (APS) de acordo com as Diretrizes Brasileiras de Hipertensão Arterial, as etapas do Processo de Enfermagem (PE) e a comunicação clínica.

## Método

A redação deste estudo seguiu o *checklist Strengthening the Reporting of Observational Studies in Epidemiology* (STROBE)^15^.

### Delineamento e local do estudo

Trata-se de estudo observacional analítico transversal, realizado a partir de dados secundários de um estudo multicêntrico, intitulado “Habilidades de prática avançada na gestão do cuidado em consultas de enfermagem na Atenção Primária à Saúde: diagnóstico situacional e proposta de capacitação”, que avalia diferentes linhas de cuidado para enfermeiros na APS por meio da gravação de 203 consultas de enfermagem realizadas por 32 enfermeiros. A coleta de dados ocorreu em 17 Unidades Básicas de Saúde (UBS) de quatro unidades federativas diferentes, sendo 6 localizadas na região Norte, 7 no Nordeste e 4 no Sudeste do país.

No presente estudo foram incluídas as consultas de enfermagem realizadas a pessoas que autodeclararam ter HAS e que tenham sido gravadas na íntegra (n=29), realizadas por 13 enfermeiros. 

### População e definição da amostra

O processo amostral foi definido por conveniência, considerando a inclusão de diferentes cenários do território brasileiro (Figura 1). Assim, diante da dimensão continental do país, optou-se por contemplar três das cinco macrorregiões, em cidades com perfil demográfico, de morbimortalidade e de oferta de serviços distintos, sendo duas cidades de grande e duas de pequeno porte.

**Figura 1:** Fluxograma referente ao processo de amostragem

### Critérios de seleção

Os participantes deste estudo foram enfermeiros e usuários presentes na consulta de enfermagem gravada. Os critérios de inclusão para os enfermeiros foram atuar no cuidado direto aos usuários da APS há pelo menos um ano. Para os usuários, foi considerado critério de inclusão aqueles que buscaram atendimento por alguma enfermeira que tivesse aceitado participar do estudo. 

Os enfermeiros e usuários que aceitaram participar do estudo assinaram o Termo de Consentimento Livre e Esclarecido, o termo de autorização de imagem e voz e o instrumento de caracterização demográfica.

### Coleta de dados

A coleta de dados do estudo multicêntrico ocorreu no período de maio a julho de 2022, após convite e pactuação com a gestão e enfermeiros participantes. Os dados foram coletados por duas pesquisadoras. À primeira atribuiu-se o convite aos usuários na sala de espera e a obtenção do consentimento. Realizou-se, ainda, a caracterização do usuário, por meio de dados sociodemográficos, motivo da procura da Consulta de Enfermagem e antecedentes de saúde, assim como de enfermeiros acerca de dados sociodemográficos, da formação, características do campo e motivação profissional. A segunda pesquisadora foi responsável por manipular as duas câmeras de filmagem, modelo GoPro^®^ Hero9, sendo uma posicionada no consultório e uma no corpo da enfermeira, fixada por meio do uso de faixa peitoral elástica.

Além da gravação, os dados sobre os registros das consultas foram extraídos de prontuários físicos ou eletrônicos após o término do atendimento. A obtenção dos dados ocorreu por meio de um instrumento elaborado pelas pesquisadoras, construído com o objetivo da avaliação do registro do processo de enfermagem nos prontuários. Este processo permitiu avaliar as etapas do processo de enfermagem e o raciocínio clínico não expressado verbalmente aos pacientes.

Adicionalmente, no presente estudo, no período de setembro a outubro de 2023, foram extraídos os dados das gravações de consultas de enfermagem às pessoas com HAS, com enfoque na avaliação das recomendações contidas nas Diretrizes Brasileiras de HAS, a partir das etapas do PE.

### Variáveis do estudo e instrumentos de coleta de informações

Para avaliação das Consultas de Enfermagem em relação ao cuidado do usuário com HAS, foi elaborado um *checklist*. Tal documento teve como referência as Diretrizes Brasileiras de Hipertensão Arterial^4^ para embasamento da avaliação de parâmetros clínicos; a Resolução COFEN (Conselho Federal de Enfermagem) 736/2024^10^ e o Manual Processo de Enfermagem na Atenção Primária à Saúde^16^ para a avaliação do cumprimento das etapas do Processo de Enfermagem, além do guia de habilidades de comunicação *Calgary- Cambridge Guide to the Medical Interview - Communication Process* ^17^ para avaliação das estratégias de comunicação clínica utilizadas.

O processo de construção dos itens e subitens do *checklist* aconteceu com a avaliação conjunta das autoras do artigo, uma equipe composta por enfermeiras e nutricionistas, com vasta experiência em pesquisa e no cuidado aos usuários que apresentam condições crônicas na APS. A discussão dos conceitos e a integração das questões que compõem o *checklist* foram feitas em reuniões em que participaram no mínimo três pesquisadoras. Após a obtenção de consenso, foi realizada uma análise-piloto de duas consultas de enfermagem a fim de calibrar o instrumento e padronizar as avaliadoras em conceitos e respostas.

A partir dos resultados do piloto, foram discutidas questões que apresentavam divergência entre as respostas das avaliadoras a fim de que, ao final, um novo consenso fosse criado. Nestas reuniões, além da análise do conteúdo, foram retiradas perguntas, inseridas novas indagações e reformuladas outras que apresentaram tal necessidade. Esse processo resultou em um encontro semanal de 1h30 por três semanas. Após concordância, deu-se início ao processo de avaliação do total das consultas de enfermagem por duas enfermeiras (LC e CD).

Os itens e subitens do *checklist* foram organizados nas seções: “Avaliação de Enfermagem”, “Diagnóstico de Enfermagem”, “Planejamento de Enfermagem”, “Implementação da Assistência de Enfermagem”, “Evolução de Enfermagem”, “Comunicação” e “Caracterização da Consulta”. Para as análises, a caracterização da consulta não foi considerada como uma etapa do índice de cumprimento das etapas do PE e comunicação. Os subitens são questões dependentes dos itens, por exemplo: para que a pergunta “Profissional realizou técnica correta de aferição de pressão arterial?” fosse aplicável, era necessário que a resposta para a pergunta “Profissional realizou aferição de pressão arterial?” fosse “sim”. Da mesma maneira, para que a pergunta “Profissional solicitou exame de glicemia de jejum?” fosse aplicável, era necessário que a resposta para a pergunta “Profissional solicitou exames complementares básicos?” tivesse sido “sim”.

Cada item e subitem foi avaliado como presente ou ausente segundo as ações da enfermeira durante a consulta. Adicionalmente, foram incluídas colunas de evidência de cena e observações, para preenchimento caso houvesse necessidade. Na coluna de “Evidência de Cena”, foi inserida a correspondente parte do vídeo em que a resposta ficava evidente, bem como o minuto em que ela aparecia. O campo de “Observações” ficou aberto para escrita livre de observações que a avaliadora achasse pertinente. Subsequentemente, deu-se início ao processo oficial de análise dos vídeos, que levou cerca de 2 meses para ser finalizado na íntegra.

Tanto a construção do instrumento para avaliação dos registros de prontuário e do *checklist* para análise das consultas de enfermagem gravadas, quanto o gerenciamento dos dados foram realizados em formato eletrônico, na plataforma RedCap (*Research Electronic Data Capture*).

Para reduzir o viés de seleção, foram definidos critérios de inclusão e exclusão. O instrumento de análise das consultas de enfermagem foi construído tendo como base referências de uso nacional no campo do cuidado à pessoa com HAS na APS, reduzindo possíveis vieses de mensuração, bem como o treinamento uniforme das pesquisadoras que analisaram os vídeos das consultas de enfermagem. Além disso, a transparência na descrição dos procedimentos e as análises bivariadas empregadas ajudam a avaliar a robustez dos resultados. Essas medidas, em conjunto, fortalecem a qualidade metodológica e a credibilidade das conclusões do presente estudo.

### Tratamento e análise dos dados

Realizou-se inicialmente uma análise descritiva univariada de todas as variáveis, utilizando medidas de tendência central (média ou mediana) e de dispersão (desvio-padrão ou intervalo interquartílico), conforme apropriado, além de distribuições de frequência para variáveis categóricas. A normalidade da distribuição de cada variável contínua foi formalmente avaliada por meio do teste de Shapiro-Wilk. Para a análise bivariada com o intuito de explorar a relação entre o cumprimento percentual das etapas do PE e o tempo de duração da consulta, optou-se por uma abordagem gráfica complementada por análise estatística inferencial. Especificamente, foi construído um gráfico de dispersão com três elementos:

1. pontos representando cada observação,
2. retas de regressão linear tracejadas para visualizar a tendência da relação em cada subgrupo e
3. distinção por cores baseada na variável categórica “Número de Diagnósticos Reportados”. Considerando que a variável ‘tempo de duração da consulta’ violou o pressuposto de normalidade (Shapiro-Wilk, p < 0,05), a força e a direção da associação contínua entre essas duas variáveis foram quantificadas utilizando-se o método não paramétrico do coeficiente de correlação de postos de Spearman (ρ).

Para avaliar a adesão às Diretrizes Brasileiras de Hipertensão Arterial, foi realizada uma análise observacional transversal, utilizando o PE como estrutura metodológica de referência. Um *checklist* de extração de dados foi construído alinhando cada etapa do processo - Avaliação, Diagnóstico, Planejamento, Implementação e Evolução de enfermagem - com as recomendações específicas das Diretrizes. Por meio da observação direta de 29 consultas, verificou-se a execução de itens críticos para o manejo da HAS, como a técnica correta de aferição de pressão arterial, a investigação de fatores de risco cardiovascular, a realização do exame físico direcionado, a educação em saúde e o estabelecimento de um plano de cuidado compartilhado. Dessa forma, o PE serviu como uma lente analítica para mensurar de forma sistemática e integral se as práticas observadas correspondiam ao padrão de excelência clínica preconizado.

O percentual de cumprimento das etapas do PE foi calculado com base no número de itens analisados, considerando que todas as etapas possuem o mesmo peso. Para isso, foi determinado o total de itens que compõem integralmente o PE, garantindo uma distribuição equitativa entre as etapas. Esses percentuais foram descritos e relacionados graficamente segundo as características dos usuários, dos profissionais de enfermagem e as características das consultas. Todas as análises estatísticas foram realizadas usando o *software* estatístico R versão 4.1.3 (*R Project for Statistical Computing*) junto com o *software* estatístico R Studio versão 1.4.1717 (RStudio).

### Considerações éticas

Os preceitos éticos e legais foram cumpridos, respeitando-se a Resolução n.º 466/2012 do Conselho Nacional de Saúde. O estudo foi aprovado pelo Comitê de Ética em Pesquisa (56255622.2.0000.0071) e teve anuência dos centros coparticipantes. Em conformidade com a Ciência Aberta, os dados estão disponíveis a partir de consulta aos autores, de forma parcial, uma vez que a gravação das consultas de enfermagem pode identificar os enfermeiros, usuários e unidades de saúde participantes.

## Resultados

### Usuários

No presente estudo, foram incluídas 29 gravações de consultas de enfermagem concernentes aos usuários das unidades de Atenção Primária à Saúde que autorreferiram diagnóstico de HAS. Os usuários incluídos nas análises apresentaram uma média de idade de 56,62 anos (± 15,28), sendo 86,21% do sexo feminino e 68,96% pretos ou pardos.

Em relação às condições socioeconômicas, 72,41% dos usuários recebiam alguma renda e, destes, 75,87% recebiam entre 0 e 2 salários-mínimos. No que tange às localidades de origem, três usuários eram do município localizado no estado de Alagoas (AL), 11 do estado do Amazonas (AM), seis do Rio Grande do Norte (RN) e nove do estado de São Paulo (SP). Ademais, 82,76% referiram morar acompanhados de familiares. Além de hipertensão arterial, 55,27% dos usuários referiram ter mais de três diagnósticos diferentes. Os principais motivos de procura do serviço de saúde foram evento agudo (30,37%), coleta de exame citopatológico de colo uterino (17,24%) e avaliação de resultado de exame (10,34%).

### Enfermeiras

Um total de 13 enfermeiros foi responsável pelas consultas aos usuários com HAS, sendo 1 de AL, 4 de AM, 3 de RN e 5 de SP. Destes, 76,92% eram do sexo feminino. Os serviços de saúde nos quais trabalhavam eram, em 61,54% dos casos, Unidades Básicas de Saúde com Estratégia Saúde da Família e, dos 13 profissionais participantes do estudo, 8 (61,5%) possuíam pelo menos 5 anos de experiência na área de atuação. Todos os profissionais afirmaram possuir algum tipo de pós-graduação e, deles, 91,54% tinham residência ou especialização de APS, em Saúde da Família e Comunidade, em Saúde Coletiva ou em Saúde Pública.

No que diz respeito aos elementos facilitadores ou dificultadores para a realização das consultas, 76,92% dos enfermeiros possuíam sala exclusiva e 61,54% tinham acesso a alguma Taxonomia como norteadora de Diagnósticos de Enfermagem. Demais informações acerca das características dos enfermeiros cujas consultas foram avaliadas neste estudo encontram-se dispostas na Tabela 1. Como ilustrado ainda nesta tabela, houve casos em que os enfermeiros não informaram ou relataram a participação em mais de um curso no último ano e a aplicação de múltiplos protocolos no atendimento aos usuários.

**Tabela 1:** Perfil profissional de atuação e de formação dos enfermeiros atuantes nos serviços de saúde (n = 13). Brasil, 2022-2023

Variáveis	% (n)*
**Salário** 1 a 2 SM^†^ 3 a 4 SM^†^ 5 a 6 SM^†^ > 7 SM^†^	15,38 (2) 23,08 (3) - 61,54 (8)
**Outro vínculo empregatício** Sim Não	38,46 (5) 61,54 (8)
**Tipo de equipe na Unidade** Estratégia Saúde da Família UBS^‡^ Mista UBS^‡^ Tradicional UBS^‡^ Rural	61,54 (8) 15,38 (2) 15,38 (2) 7,69 (1)
**Existe Equipe Multidisciplinar na Unidade** Sim Não	61,54 (8) 38,46 (5)
**Tempo de experiência como enfermeiro da APS** ^§^ Entre 1 e 5 anos Entre 6 e 10 anos > 10 anos	3,46 (5) 46,15 (6) 15,38 (2)
**Existem dificuldades para realização de consulta** Sim Não	69,23 (9) 30,77 (4)
**Instrumentos padronizados sobre Processo de Enfermagem** Sim Não	38,46 (5) 61,54 (8)
**Taxonomia utilizada para Diagnóstico de Enfermagem** NANDA^||^ CIPE^¶^ Outros Nenhuma	23,08 (3) 30,77 (4) 7,69 (1) 38,46 (5)
**Motivação profissional** Com satisfação Sem satisfação	76,92 (10) 23,08 (3)
**Residência ou Especialização em APS** ^§^ Sim Não	30,77 (4) 69,23 (9)
**Especialização em Saúde da Família e Comunidade, Saúde Coletiva ou Saúde Pública** Sim Não	61,54 (8) 38,46 (5)
**Número de Profissionais com Mestrado** Sim Não	30,77 (4) 69,23 (9)
**Participação em cursos no último ano** Saúde do Idoso Cuidado aos crônicos (HAS**/DM^††^) Processo de Enfermagem	23,08 (3) 30,77 (4) 30,77 (4)
**Teve aulas sobre o Processo de Enfermagem** Sim Não Não sei	84,62 (11) 7,69 (1) 7,69 (1)
**Na sua unidade usam Protocolos de Enfermagem** Sim Não	46,15 (6) 53,85 (7)
**Protocolos usados na Saúde do Idoso** Caderno de Atenção Básica do Ministério da Saúde Protocolo do Estado Protocolo de outros municípios Protocolo do COREN^‡‡^ Protocolo da sua secretaria municipal de saúde	76,92 (10) 7,69 (1) 7,69 (1) - 15,38 (2)
**Protocolos usados para atenção aos Crônicos** Caderno de Atenção Básica do Ministério da Saúde Protocolo Ministério da Saúde Protocolo do Estado Protocolo de outros municípios Protocolo do COREN^‡‡^ Protocolo da sua Secretaria Municipal de Saúde	76,92 (10) 61,54 (8) 23,08 (3) 7,69 (1) - 15,38 (2)

*%(n) = Valor percentual (número bruto); ^†^SM = Salário-mínimo, correspondente a R$1518,00 no Brasil, ano 2025; ^‡^UBS = Unidade Básica de Saúde; ^§^APS = Atenção Primária à Saúde; ^||^NANDA = *North American Nursing Diagnosis Association*; ^¶^CIPE = Classificação Internacional para a Prática de Enfermagem; **HAS = Hipertensão Arterial Sistêmica; ^††^DM = Diabetes Mellitus; ^‡‡^COREN = Conselho Regional de Enfermagem

O tempo médio de cada consulta foi de 22,89 minutos (± 13,54), 66,67% delas foram interrompidas durante a realização, 55,17% foram agendadas previamente e a queixa do usuário estava relacionada à HAS em 24,14% dos atendimentos.

A descrição a seguir trata do conteúdo gerado a partir da análise dos vídeos das consultas, de acordo com o *checklist*. Na etapa de Avaliação de Enfermagem, 72,41% dos profissionais não aferiram a pressão arterial durante o atendimento e, dos que aferiram, 25% realizaram tal procedimento com técnica correta. A busca por dados antropométricos esteve ausente em 51,72% das consultas, assim como a ausculta cardíaca (86,21%). A avaliação das extremidades dos usuários esteve presente em 10% das consultas, em que os profissionais investigaram a possibilidade da presença de edema.

Os profissionais realizaram avaliação de exames complementares básicos em 31,0% das consultas e em 88,89% delas o resultado foi comunicado ao usuário. O uso de medicamentos contínuos foi investigado em 51,72% das consultas e em nenhuma delas questionou-se a respeito de uso de medicamentos sem prescrição e/ou uso de drogas ilícitas.

Os fatores de risco específicos para doença cardiovascular real foram explorados em 72,42% das consultas, sendo a idade o mais frequente (66,67%). A presença do tabagismo, consumo de álcool ou quantitativo da ingestão de sódio não foi explorada em 94,24%, 95,25% e 95,24% das consultas, respectivamente. Doença arterial coronariana e renal crônica estiveram presentes em 1 e 3 atendimentos, respectivamente. A investigação da presença de diabetes foi evidenciada em 42,86% das consultas.

Em 79,31% dos atendimentos não foram investigados aspectos psicossociais, culturais e socioeconômicos que pudessem estar associados aos valores de medidas da pressão arterial e comorbidades e, dos que continham tal investigação, o estresse emocional foi o mais frequente (66,67%).

Estiveram ausentes em 100% das consultas: avaliação de Índice Tornozelo Braquial (ITB), ausculta de artérias carótidas, cálculo de Taxa de Filtração Glomerular (TFG), exame neurológico, antecedentes de Acidente Vascular Encefálico (AVE) e investigação de retinopatia hipertensiva, cálculo de Velocidade de Onda de Pulso (VOP), avaliação de *ictus* em palpação, investigação de história familiar de início precoce de HAS, investigação de apneia do sono e de pressão de pulso.

A comunicação do Diagnóstico de Enfermagem não foi evidente em nenhuma das consultas. Em relação à etapa de Planejamento, 89,66% dos profissionais não estabeleceram metas e o planejamento foi construído em conjunto com o usuário em 20,69% dos atendimentos.

A Prescrição de Enfermagem não foi verbalizada em 75,86% das consultas e em nenhuma delas tal prescrição foi entregue por escrito ao usuário. Por fim, o cálculo do risco cardiovascular não ficou evidente em nenhum dos atendimentos. As consultas de seguimento foram 65,62% dos atendimentos e a etapa de Evolução de Enfermagem não ficou explícita em nenhum deles.

No que tange às habilidades de comunicação e relacionamento interpessoal dos profissionais, 65,52% realizaram identificação do usuário e 86,21% não se apresentaram. O conforto e a privacidade foram garantidos em 96,55% das consultas e a entrevista foi iniciada com questões abertas em 51,72% dos atendimentos. Ademais, 79,31% dos profissionais mantiveram contato visual com o usuário durante a interação e ofereceram tempo suficiente para o usuário se expressar, sem interrupções.

Além disso, 58,62% dos profissionais realizaram escuta ativa e 93,1% utilizaram linguagem adaptada às condições educacionais e socioeconômicas dos usuários. A verificação da compreensão da pessoa e o encorajamento ao esclarecimento de dúvidas estiveram presentes em 79,31% dos atendimentos e a técnica de sumarização foi aplicada em 55,17% dos encontros clínicos. Ainda, 75,86% dos usuários não foram envolvidos no planejamento de opções terapêuticas a respeito do próprio processo saúde-doença-cuidado e não foi solicitada devolutiva do usuário a respeito do atendimento prestado em 89,66% das consultas.

### Processo de Enfermagem

*Segundo a execução*

Na Tabela 2 é demonstrado que, em resumo, do total dos 48 itens avaliados pelo *checklist*, verificou-se que a mediana de itens cumpridos foi de 18, o que indica que 50% dos enfermeiros cumpriram até 18 itens do Processo de Enfermagem. Esse valor corresponde a uma mediana de 20% de cumprimento em relação ao total de etapas do PE.

**Tabela 2:** Distribuição do cumprimento das etapas do Processo de Enfermagem, segundo itens propostos pelas Diretrizes Brasileiras de Hipertensão Arterial (N = 29). Brasil, 2022-2023

Variáveis	Sim % (n)*	Não % (n)*
**Avaliação de Enfermagem realizada pelos profissionais**		
Aferiu a pressão arterial durante a consulta?	27,59 (8)	72,41 (21)
Buscou dados antropométricos do usuário?	48,28 (14)	51,72 (15)
Realizou ausculta de artérias carótidas?	-	100,0 (29)
Realizou ausculta cardíaca?	13,79 (4)	86,21 (25)
Contou frequência cardíaca?	3,45 (1)	96,55 (28)
Examinou extremidades?	10,34 (3)	89,66 (26)
Realizou avaliação de ITB^†^?	-	100,0 (29)
Verificou resultados de exames complementares básicos relacionados à hipertensão ou RCV^‡^?	31,03 (9)	68,97 (20)
Avaliou função renal por meio de cálculo de TFG^§^ com CKD-EPI^||^ ou MDRD^¶^?	-	100,0 (29)
Examinou abdômen?	6,9 (2)	93,1 (27)
Realizou exame neurológico?	-	100,0 (29)
Investigou medicamentos em uso contínuo?	51,72 (15)	48,28 (14)
Investigou uso de medicamentos sem prescrição médica?	-	100,0 (29)
Investigou uso de drogas ilícitas?	-	100,0 (29)
Investigou fatores de risco específicos para doença cardiovascular real?	72,41 (21)	27,59 (8)
Investigou fatores que podem modificar o risco do usuário hipertenso?	27,59 (8)	72,41 (21)
Investigou aspectos psicossociais, culturais e socioeconômicos que podem estar interferindo nos valores aferidos/nas comorbidades?	20,69 (6)	79,31 (23)
Realizou avaliação de VOP**?	-	100,0 (29)
Avaliou *ictus* em palpação?	-	100,0 (29)
**Diagnóstico de Enfermagem**		
Elencou diagnóstico(s) de enfermagem:	-	100,0 (29)
Comunicou diagnóstico(s) elencado(s) ao usuário?	-	100,0 (29)
**Planejamento de Enfermagem**		
Determinou resultados que se espera alcançar e/ou estabeleceu metas?	10,34 (3)	89,66 (26)
Estabeleceu ações ou intervenções de enfermagem que serão realizadas para o alcance das metas?	33,33 (1)	66,67 (2)
**Implementação da Assistência de Enfermagem**		
Usuário tem necessidade de solicitação de exames complementares básicos?	17,24 (5)	82,76 (24)
Realizou prescrição de enfermagem? (Seja escrita ou verbal)	24,14 (7)	75,86 (22)
Dos que realizaram prescrição (n=7), esta foi entregue ao usuário escrita no papel?	-	100,0 (7)
**Evolução de Enfermagem**		
Avaliou a assistência de enfermagem?	-	100,0 (29)
Elencou novos diagnósticos de enfermagem para a necessidade que está sendo apresentada?	-	100,0 (29)

*%(n) = Valor percentual (número bruto); ^†^ITB = Índice Tornozelo-Braquial; ^‡^RCV = Risco Cardiovascular; ^§^TFG = Taxa de Filtração Glomerular; ^||^CKD-EPI = *Chronic Kidney Disease Epidemiology Collaboration*, equação para estimar a taxa de filtração glomerular; ^¶^MDRD = *Modification of Diet in Renal Disease*, fórmula para estimar a taxa de filtração glomerular; **VOP = Velocidade de Onda de Pulso

Conforme ilustrado na Figura 2, observou-se uma correlação positiva e estatisticamente significativa entre um maior tempo de duração da consulta de enfermagem e a maior adesão às etapas do Processo de Enfermagem (PE) em pacientes com HAS. A análise do tempo de consulta pelo teste de Shapiro-Wilk confirmou a ausência de normalidade em sua distribuição (p = 0,0121). Diante desse resultado, optou-se pela utilização do teste não paramétrico de Spearman para avaliar a correlação.

**Figura 2:** Correlação entre o tempo de duração da consulta de enfermagem e o cumprimento das etapas do Processo de Enfermagem junto aos usuários com Hipertensão Arterial na Atenção Primária à Saúde. Brasil, 2022-2023

A análise estratificada por complexidade diagnóstica (Figura 3) revelou um gradiente de magnitude de correlação, embora sem significância estatística em nenhum dos subgrupos (p > 0,05). Especificamente, observou-se uma correlação positiva moderada (ρ = 0,521; p = 0,150) no grupo com apenas HAS, uma correlação positiva muito fraca (ρ = 0,138; p = 0,653) no grupo com um diagnóstico adicional e uma correlação positiva de magnitude forte (ρ = 0,685; p = 0,090) no grupo com maior complexidade (2-3 diagnósticos adicionais). Este padrão ascendente sugere que a relação entre tempo de consulta e adesão ao PE intensifica-se progressivamente com o aumento da complexidade do quadro clínico.

**Figura 3:** Correlação entre os diagnósticos associados à Hipertensão Arterial e o tempo de consulta necessário para o cumprimento das etapas do Processo de Enfermagem. Brasil, 2022-2023

*Segundo o registro*

De acordo com as etapas do PE, 79,31% dos atendimentos foram descritos em prontuário e o conteúdo era legível em 87,5% dos registros. Na Avaliação, os dados subjetivos estavam ausentes em 52,17% das consultas e os dados objetivos estavam ausentes em 86,96% delas. 73,91% das consultas não apresentaram registro do diagnóstico de enfermagem e 95,65% delas continham pelo menos um diagnóstico segundo a Classificação Internacional de Atenção Primária (CIAP).

Em relação à etapa do Planejamento, em 95,65% das consultas não houve registro de metas e, das que apresentaram, uma delas tinha de fato relação com o diagnóstico de enfermagem elencado anteriormente. A prescrição de enfermagem esteve presente em dois registros de consultas, que estavam condizentes com as metas e diagnósticos de enfermagem previamente estabelecidos. A etapa de Implementação esteve ausente em 95,65% dos registros e a etapa de Evolução de Enfermagem ficou evidente em 50% deles.

## Discussão

Os resultados deste estudo indicam uma lacuna significativa entre a prática observada na Atenção Primária à Saúde e os padrões de cuidado recomendados pelas Diretrizes Brasileiras de Hipertensão Arterial, quando analisados através da lente do Processo de Enfermagem (PE). A baixa frequência de realização de componentes essenciais da avaliação - como a aferição técnica da pressão arterial, a investigação de fatores de risco modificáveis (como ingestão de sódio e sedentarismo) e o exame físico direcionado - sugere que o cuidado está sendo realizado de forma mecanizada e fragmentada, não aproveitando a consulta como uma oportunidade para uma captação completa e precisa do perfil de risco cardiovascular do usuário. 

A quase ausência da formulação de diagnósticos de enfermagem (100% não elencaram) é particularmente preocupante, pois demonstra que o julgamento clínico não está sendo formalizado, impedindo que o planejamento de cuidado seja específico, individualizado e baseado nas reais necessidades de saúde identificadas. Tal fragilidade revelou-se desde o momento de Avaliação de Enfermagem, que acontece de maneira incompleta. Consequentemente, considerando que as etapas do PE são inter-relacionadas e interdependentes, a realização das etapas subsequentes fica comprometida. Tal resultado condiz com achados de estudo que realizou diagnóstico situacional da atuação do enfermeiro na APS de Santa Catarina^18^ realizado em 2023, que chegou à conclusão de que a aplicação do PE nas consultas de enfermagem ainda se dá de maneira incipiente e fragmentada.

A desconexão entre a coleta de dados e as etapas subsequentes do PE resulta em um ciclo de cuidado incompleto. A ausência de diagnósticos clínicos sistematizados compromete o planejamento, que tende a ser inespecífico ou ausente, como evidenciado pela baixa taxa de estabelecimento de metas conjuntas (10,34%). Esse cenário já foi descrito em outros contextos, nos quais a elaboração de planos de cuidado individualizados mostrou-se um diferencial na prática assistencial, favorecendo adesão e controle da hipertensão^19^ ^-^ ^20^. Consequentemente, as intervenções de enfermagem implementadas carecem de base teórica e de direcionamento claro, centrando-se em solicitações de exames e prescrições genéricas, em detrimento de estratégias educativas e comportamentais. No entanto, evidências robustas apontam que intervenções educativas lideradas por enfermeiros reduzem de forma significativa os níveis pressóricos e favorecem o autocuidado^8^ ^,^ ^21^ ^-^ ^22^.

A alta proporção de profissionais que não forneceram *feedback* ou não verificaram a compreensão do usuário (89,66%) reforça uma comunicação unidirecional, que não promove corresponsabilização. Estudos qualitativos também evidenciam que, na prática, a Consulta de Enfermagem tende a ser pouco criativa e centrada no profissional, limitando a participação ativa do usuário^23^. Dessa forma, observa-se que a lacuna na integração entre diagnóstico, planejamento e intervenção repercute diretamente na efetividade do cuidado em condições crônicas, reforçando a necessidade de reorganização do processo de trabalho para garantir cuidado centrado no usuário e baseado em evidências.

Com o intuito de analisar os fatores relacionados à aplicação do PE em sua totalidade, foram consideradas as características dos usuários atendidos, dos enfermeiros e das consultas avaliadas. Um importante aspecto foi o tempo de consulta, pois ficou evidente que pessoas com múltiplas comorbidades necessitam de um tempo de consulta maior para que todas as necessidades de saúde sejam respondidas. Além disso, houve interrupção na maioria dos atendimentos, o que desorganiza o raciocínio clínico e traz prejuízo no manejo do tempo, evidenciado por uma revisão integrativa de literatura realizada em 2020^24^.

Esbarra-se, então, no contexto da comunicação clínica, ponto importante na consulta de enfermagem, porém avaliado neste estudo como ainda sendo algo secundário. Ações como comunicar os diagnósticos e as metas terapêuticas, entregar a prescrição de enfermagem por escrito e avaliar a assistência junto ao usuário são essenciais para a comunicação clínica efetiva^25^ ^-^ ^26^, para a adesão ao tratamento^27^ e melhoria do vínculo e da satisfação^28^. Entretanto, manejar a agenda e lidar com as interferências externas que acabam sendo responsabilidade dos enfermeiros na APS ainda é um desafio para a plena implementação do PE e para a execução da consulta de enfermagem que contempla todos os itens recomendados pelas Diretrizes de HAS^29^. 

Análise de consultas de enfermagem realizadas no Rio de Janeiro em 2021 evidenciou profissionais sobrecarregados com a pressão assistencial de agendas lotadas, que gerou como resultado um modelo de atendimento médico-centrado e focado principalmente em queixa-conduta^30^, o que dificulta a utilização do PE em sua integralidade, bem como pode interferir negativamente com desfechos clínicos dos usuários.

Destaca-se o não cumprimento da etapa de Diagnóstico de Enfermagem. Em nosso estudo, essa etapa esteve presente 26,09% das consultas avaliadas, e justifica tal fato com a falta de habilidade e manejo com as taxonomias classificatórias. Todavia, 38,5% dos profissionais avaliados referiram não utilizar nenhuma taxonomia para diagnóstico de enfermagem. Tais evidências dialogam com resultados em outros contextos, cuja dificuldade em implementar a etapa de diagnósticos no PE ainda é fortemente marcada por complexidade teórica, lacunas na formação, falta de apoio institucional, alta carga de trabalho e baixo domínio prático^31^ ^-^ ^32^.

Embora a maioria dos profissionais relate ter tido formação na graduação sobre Processo de Enfermagem e possua especialização e/ou residência, verifica-se que há um distanciamento entre teoria e a real utilização dessa ferramenta por eles, sendo o principal fator dificultador a falta de capacitação e a implementação do conhecimento na prática^33^, entendendo-se que o PE é uma ferramenta para a prática segura do enfermeiro. Tem-se, portanto, que conhecer as Taxonomias que padronizam os cuidados específicos da enfermagem, assim como padrões de cuidado em Programas de Saúde, pois protocolos gerais, por exemplo, não garantem a completa apropriação do PE na realização do encontro clínico durante a consulta.

Desta forma, ainda que muitos profissionais participantes do estudo tenham referido utilizar algum protocolo para nortear o atendimento, mais da metade deles não era específica para a enfermagem, o que pode configurar um empecilho para a consolidação de um PE qualificado^34^. O uso de protocolos de enfermagem é fundamental, pois auxiliam na tomada de decisão com foco na segurança do paciente e respaldam o profissional considerando os princípios éticos da profissão^35^. A necessidade da implementação de protocolos como estratégias de redução dos dificultadores e como fortalecimento do processo^36^ teria grande importância, visto que 53,85% dos enfermeiros relataram não utilizar Protocolos de Enfermagem durante sua prática assistencial.

Em municípios de grande porte do interior do estado de São Paulo^37^, em 2015, o PE em uma unidade de APS ainda era implementado de forma incipiente, sendo que 19% dos enfermeiros referiram não utilizar, 38% avaliaram que utilizam raramente e 29% algumas vezes. Em comparação aos nossos resultados, houve melhora na utilização do PE, ainda que não contemple as suas cinco etapas. Assim, é fundamental que, associado à elaboração de diretrizes e protocolos clínicos, seja calculado e evidenciado o tempo necessário para o cumprimento das atividades previstas, pois isso poderia implicar até mesmo no grau de recomendação de determinada intervenção, na priorização de casos mais graves e na necessidade do aumento do quantitativo de profissionais disponíveis ao atendimento a esses usuários^38^. 

Apesar dos desafios para o PE na APS durante o cuidado junto à pessoa com HAS, as consultas de enfermagem são práticas assistenciais que favorecem o cuidado longitudinal, uma vez que as intervenções são pautadas em evidências científicas e direcionam-se aos aspectos clínicos, sociais, culturais e regionais do cuidado, de forma personalizada^39^ ^-^ ^41^.

A partir do PE, pautado em teorias e utilizado em sua totalidade, desenvolvido junto com a pessoa, em âmbito individual, há maior satisfação com o acompanhamento de saúde, o que favorece mudanças de comportamentos e melhora da situação clínica e da qualidade de vida^42^ ^-^ ^43^. Para os profissionais, a consulta de enfermagem, apesar dos desafios, aumenta a autonomia da enfermeira e produz experiências positivas de atuação^44^ ^-^ ^45^. Na esfera das políticas públicas e do Sistema Único de Saúde (SUS), a APS poderá ser ainda mais fortalecida, com expressiva contribuição da enfermagem para a redução da carga de doenças crônicas não transmissíveis.

Este estudo tem algumas limitações. Primeiro, não foi calculado o tamanho da amostra e a seleção das unidades ocorreu por conveniência. No entanto, foram incluídas unidades de 4 diferentes municípios do país que apresentam características sociodemográficas particulares das regiões do Brasil. A segunda limitação faz referência ao número de consultas analisadas (n=29) e ao número de Enfermeiros (n=13), resultando em características heterogêneas dos profissionais e um limitado espectro. Pode ser uma limitação o possível viés de informação relacionado ao instrumento utilizado para a avaliação das consultas de enfermagem. 

Finalmente, como acontece nos estudos observacionais, existe a possibilidade de um viés de seleção atribuível aos enfermeiros que aceitaram participar, que podem apresentar práticas diferentes em relação aos que recusaram participar da pesquisa. Ademais, há possibilidade de viés de desejabilidade social, que pode ser desencadeado pela mudança comportamental por ser participante da pesquisa e estar sujeito à gravação e observação. Apesar das limitações, o presente estudo discute importantes resultados no que tange à avaliação de todas as etapas do PE na APS no cuidado à pessoa com HAS. Os achados contribuem para a reflexão crítica do cuidado de enfermagem, além de apontar para a importância do registro do PE. Apresenta os fatores associados aos desafios da utilização do PE, o que guiará ações e pesquisas e permitirá adequação de documentos e políticas públicas de modo a contemplar as especificidades da enfermagem na APS.

Devido à quantidade ainda escassa de estudos realizados no Brasil sobre tal temática, essa pesquisa, enquanto estudo primário, abre caminhos e alumia futuras discussões para produção de instrumentos e conhecimentos que fortaleçam a prática clínica de enfermagem. Pretende-se, desta forma, impactar positivamente a razão de existir enquanto categoria profissional: proporcionar o melhor cuidado aos usuários, fundamentado em instrumentos sólidos e baseado nas melhores evidências científicas.

## Conclusão

Evidenciou-se fragilidade na avaliação dos elementos recomendados pelas Diretrizes Brasileiras de Hipertensão Arterial e na consolidação do PE durante as consultas de enfermagem às pessoas com HAS. Complexidade dos usuários, tempo e interrupção de consultas, elevada demanda assistencial, formação dos profissionais e falta de protocolos de enfermagem estruturados a partir das etapas do PE foram hipóteses levantadas para justificar tal achado. Além disso, a publicação de diretrizes assistenciais sem documento norteador para sua aplicabilidade consiste em empecilho para sua real implementação no cotidiano.

## Data Availability

Todos os dados gerados ou analisados durante este estudo estão incluídos neste artigo publicado.

# Translation: Análisis de consultas de enfermería a personas con hipertensión arterial sistémica en la Atención Primaria de Salud^*^

**DOI:** 10.1590/1518-8345.7998.4875

**Authors:** Luciana Assis Couto, Carin Caroline Dzembatyi, Dafne Louize Gomes Fernandes, Daiana Bonfim, Andressa Teoli Nunciaroni, Andrea Liliana Vesga Varela

**Affiliations:** 1 Hospital Israelita Albert Einstein, São Paulo, SP, Brasil.; 2 Centro Universitário Campo Real Irati, Irati, PR, Brasil.; 3 Sociedade Beneficente Israelita Brasileira Albert Einstein, São Paulo, SP, Brasil.; 4 Universidade Federal do Estado do Rio de Janeiro, Departamento de Enfermagem de Saúde Pública, Rio de Janeiro, RJ, Brasil.; 5 Scholarship holder at the Fundação de Amparo à Pesquisa do Estado do Rio de Janeiro (FAPERJ), Brasil.

**Keywords:** Hipertensión, Atención Primaria de Salud, Enfermería de Consulta, Proceso de Enfermería, Protocolos Clínicos, Atención de Enfermería.

## Abstract

**Objetivo:**

analizar la consulta de enfermería a personas con hipertensión arterial sistémica en la atención primaria de salud según las Directrices Brasileñas sobre Hipertensión Arterial, las etapas del proceso de enfermería y la comunicación clínica.

**Método:**

un estudio analítico transversal que evaluó 29 consultas de enfermería realizadas por 13 enfermeras con usuarios con hipertensión arterial en cuatro estados brasileños. La evaluación de las consultas se realizó mediante una lista de verificación estructurada. Los datos se sometieron a análisis descriptivo y bivariado, utilizando tablas y gráficos para caracterizar la muestra y explorar posibles asociaciones entre las variables.

**Resultados:**

hubo baja adhesión al Proceso de Enfermería y a las recomendaciones de las Directrices Brasileñas sobre Hipertensión. Solo se cumplieron completamente el 20% de las etapas del proceso de enfermería. Específicamente en relación con las Directrices, se observó que las acciones fundamentales eran insuficientes, como la medición de la presión arterial, la evaluación de factores de riesgo y la comunicación clínica del diagnóstico.

**Conclusión:**

hubo debilidad en la aplicación del Proceso de Enfermería en las consultas a personas con hipertensión. Factores como la complejidad de los casos, las interrupciones, la alta demanda de trabajo, las lagunas en la formación y la ausencia de protocolos estructurados contribuyeron a estos hallazgos.

## Destacados

**(1)** Los aspectos sociales, culturales y regionales deben formar parte del proceso de enfermería. **(2)** Se cubrieron todas las etapas del proceso de enfermería y la comunicación efectiva. **(3)** Las Directrices de Hipertensión pueden guiar el proceso de enfermería en la Atención Primaria de Salud. **(4)** El proceso de enfermería a personas con hipertensión necesita mejorar en la Atención Primaria de Salud.

## Introducción

Una Atención Primaria de Salud (*Atenção Primária à Saúde* - APS) reforzada, adecuadamente dimensionada y basada en el territorio, con participación comunitaria, se asocia con sistemas de salud con mejores resultados y mayor acceso a los servicios para la población^1^ ^-^ ^2^. En Brasil, la hipertensión arterial sistémica (HAS) es uno de los factores más influyentes en la mortalidad temprana debida a enfermedades cardiovasculares (ECV) cuando se asocia con factores dietéticos, metabólicos y ambientales^3^. Así, la APS es un terreno fértil para la implementación de políticas públicas e intervenciones tempranas basadas en la evidencia, con el fin de reducir las tendencias en la incidencia y complicaciones relacionadas con la HAS.

En el seguimiento longitudinal de personas con HAS en la APS, las Directrices Brasileñas sobre Hipertensión Arterial recomiendan que la atención incluya un enfoque clínico, aspectos psicosociales, culturales y socioeconómicos que determinen la salud^4^ ^-^ ^6^. Por tanto, se destaca la importancia del manejo de la HAS en la APS, ya que este punto de la *Rede de Atenção à Saúde* (Red de Atención a la Salud) considera las condiciones que pueden determinar los resultados de salud y cardiovasculares de la población.

Así, en el contexto de la APS, las enfermeras ocupan un papel destacado en la gestión de la atención, especialmente en lo que respecta a los cambios de comportamiento y la mejora de la calidad de vida. En este escenario, los estudios demuestran que las intervenciones de enfermería en la atención a personas con HAS en la APS están asociadas con la reducción de los niveles de presión arterial, la mejora de los datos antropométricos, la adherencia al tratamiento y cambios positivos en el comportamiento^7^ ^-^ ^9^.

Entre las herramientas de trabajo de los enfermeros en la APS, destacase el Proceso de Enfermería (PE). Es un instrumento metodológico a través del cual se implementa la Consulta de Enfermería^10^ y que contribuye a la sistematización del razonamiento clínico, la identificación de necesidades sanitarias y la planificación asistencial, permitiendo un plan de atención que refuerza la coordinación asistencial, la longitudinalidad y la evaluación de los resultados de salud.

Así, la enfermera desempeña un papel importante en la provisión de una atención centrada en el usuario mediante intervenciones ante necesidades de salud, lo que permite la creación de un vínculo de confianza^11^ ^-^ ^12^. También desde esta perspectiva, una revisión emergente sobre estrategias de comunicación personalizada apunta a una relación positiva entre el cuidado de enfermería personalizado y la satisfacción/adhesión al tratamiento acordado^13^, así como una contribución favorable a la construcción de vínculos y al empoderamiento de los usuarios en el autocuidado^14^.

Aunque el PE está legalmente determinado y reconocido como el método para la implementación de la atención de enfermería, aún existen varios desafíos para su uso efectivo en la APS brasileña, tanto relacionados con su operativización como con las especificidades en la atención a personas con HAS. Así, para proporcionar subvenciones que mejoren la práctica clínica y consolidar la implementación del PE en la atención de usuarios con HAS en la APS, buscamos entender cómo han utilizado las enfermeras los elementos recomendados por las Directrices Brasileñas sobre Hipertensión Arterial en las Consultas de Enfermería, considerando las etapas del PE y las estrategias de comunicación clínica.

Por ello, el presente estudio tiene como objetivo analizar la consulta de enfermería a personas con Hipertensión Arterial Sistémica (HAS) en la Atención Primaria de Salud (APS) según las Directrices Brasileñas sobre Hipertensión Arterial, las etapas del Proceso de Enfermería (PE) y la comunicación clínica.

## Método

La redacción de este estudio siguió la lista de verificación *Strengthening the Reporting of Observational Studies in Epidemiology* (STROBE)^15^.

### Diseño y lugar del estudio

Se trata de un estudio analítico observacional transversal, basado en datos secundarios de un estudio multicéntrico, titulado “Habilidades de práctica avanzada en la gestión de cuidados en consultas de enfermería en atención primaria: diagnóstico situacional y propuesta de formación”, que evalúa diferentes líneas de atención para enfermeras en la APS mediante el registro de 203 consultas de enfermería realizadas por 32 enfermeros. La recopilación de datos se realizó en 17 Unidades Básicas de Salud (UBS) de cuatro unidades federativas diferentes, 6 ubicadas en el norte, 7 en el noreste y 4 en el sureste del país.

El presente estudio incluyó consultas de enfermería realizadas con personas que se autodeclararon tener HAS y que habían sido registradas íntegramente (n=29), realizadas por 13 enfermeras.

### Población y definición de la muestra

El proceso de muestreo se definió por conveniencia, considerando la inclusión de diferentes escenarios en el territorio brasileño (Figura 1). Así, en vista de la dimensión continental del país, se decidió incluir tres de las cinco macrorregiones, en ciudades con diferentes perfiles demográficos, morbilidad y mortalidad y la prestación de servicios, dos grandes y dos pequeñas.

**Figura 1:** Diagrama de flujo que se refiere al proceso de muestreo

### Criterios de selección

Los participantes en este estudio fueron enfermeros y usuarios presentes en la consulta de enfermería registrada. Los criterios de inclusión para las enfermeras eran trabajar en atención directa para usuarios de APS durante al menos un año. Para los usuarios, el criterio de inclusión fue aquellos que buscaron atención de una enfermera que hubiera aceptado participar en el estudio.

Los enfermeros y usuarios que aceptaron participar en el estudio firmaron el Término de Consentimiento Libre y Esclarecido, el término de autorización de imagen y voz y el instrumento de caracterización demográfica.

### Recogida de datos

La recogida de datos para el estudio multicéntrico tuvo lugar de mayo a julio de 2022, tras una invitación y acuerdo con la dirección y las enfermeras participantes. Los datos fueron recopilados por dos investigadores. La primera se atribuyó a la invitación a los usuarios en la sala de espera y a la obtención de consentimiento. También se llevó a cabo la caracterización del usuario, a través de datos sociodemográficos, motivos para solicitar la Consulta de Enfermería e historial médico, así como de los enfermeros sobre datos sociodemográficos, formación, características del campo y motivación profesional. El segundo investigador fue responsable de manipular las dos cámaras de vídeo GoPro^®^ Hero9, una situada en la consulta y otra en el cuerpo del enfermero, fijadas mediante una correa elástica para el pecho.

Además del registro, los datos de los registros de las consultas se extrajeron de registros médicos físicos o electrónicos tras finalizar la consulta. Los datos se obtuvieron mediante un instrumento desarrollado por los investigadores, diseñado con el objetivo de evaluar el registro del proceso de enfermería en los registros médicos. Este proceso permitía evaluar las etapas del proceso de enfermería y el razonamiento clínico sin expresarse verbalmente a los pacientes.

Además, en el presente estudio, de septiembre a octubre de 2023, se extrajeron datos de los registros de consultas de enfermería a personas con HAS, centrándose en la evaluación de las recomendaciones contenidas en las Directrices Brasileñas de HAS, basándose en las etapas del PE.

### Variables de estudio e instrumentos de recopilación de información

Para evaluar las consultas de enfermería en relación con la atención al usuario con HAS, se elaboró una lista de verificación. Dicho documento tomó como referencia las Directrices Brasileñas sobre Hipertensión Arterial^4^ como base para la evaluación de los parámetros clínicos; la Resolución COFEN (Consejo Federal de Enfermería) 736/2024^10^ y el Manual del Proceso de Enfermería en la Atención Primaria de Salud^16^ para evaluar el cumplimiento de las etapas del Proceso de Enfermería y la guía de habilidades de comunicación *Calgary- Cambridge Guide to the Medical Interview - Communication Process* ^17^ para evaluar las estrategias de comunicación clínica utilizadas.

El proceso de elaboración de los ítems y subítems de la lista de verificación se llevó a cabo con la evaluación conjunta de los autores del artículo, un equipo compuesto por enfermeras y nutricionistas, con amplia experiencia en investigación y cuidado de pacientes que presentan enfermedades crónicas en APS. La discusión de los conceptos y la integración de las preguntas que conforman la lista de verificación se realizaron en reuniones en las que participaron al menos tres investigadores. Tras alcanzar un consenso, se realizó un análisis piloto de dos consultas de enfermería para calibrar el instrumento y estandarizar a los evaluadores en conceptos y respuestas.

Basándose en los resultados del piloto, se discutieron las preguntas que presentaban divergencia entre las respuestas de los evaluadores para que, finalmente, se creara un nuevo consenso. En estas reuniones, además del análisis del contenido, se eliminaron preguntas, se insertaron nuevas preguntas y se reformularon otras que presentaban tal necesidad. Este proceso resultó en una reunión semanal de 1:30 durante tres semanas. Tras el acuerdo, comenzó el proceso de evaluación del número total de consultas de enfermería realizadas por dos enfermeras (LC y CD). Los ítems y subelementos de la lista de verificación se organizaron en las secciones: “Evaluación de Enfermería”, “Diagnóstico de Enfermería”, “Planificación de Enfermería”, “Implementación de la Atención de Enfermería”, “Evolución de la Enfermería”, “Comunicación” y “Caracterización de la Consulta”. Para los análisis, la caracterización de la consulta no se consideró como una etapa del índice de cumplimiento de las etapas del PE y la comunicación. Los subítems son preguntas dependientes de cada ítem, por ejemplo: para que la pregunta “¿Realizó el profesional la técnica correcta de medición de la presión arterial?” fuera aplicable, era necesario que la respuesta a la pregunta “¿Realizó el profesional la medición de la presión arterial?” fuera “sí”. Del mismo modo, para que la pregunta “¿Solicitó un profesional una prueba de glucosa en ayunas?” fuera aplicable, era necesario que la respuesta a la pregunta “¿Solicitó un profesional pruebas básicas complementarias?” fuera “sí”.

Cada ítem y subítem fue evaluado como presente o ausente según las acciones de la enfermera durante la consulta. Además, se incluyeron columnas de evidencia de escenas y observaciones, que debían completarse si era necesario. En la columna “Evidencia de la escena”, se insertó la parte correspondiente del vídeo en la que la respuesta era evidente, así como el minuto en que apareció. El campo “Observaciones” estaba abierto para la escritura libre de observaciones que el evaluador considerara pertinentes. Posteriormente, comenzó el proceso oficial de análisis de los vídeos, que tardó unos 2 meses en finalizarse por completo. Tanto la construcción del instrumento para la evaluación de historiales médicos como la lista de verificación para el análisis de consultas registradas de enfermería, así como la gestión de datos, se llevaron a cabo en formato electrónico, en la plataforma RedCap *(Research Electronic Data Capture)*

Para reducir el sesgo de selección, se definieron criterios de inclusión y exclusión. El instrumento para analizar las consultas de enfermería se construyó basándose en referencias de uso nacional en el campo de la atención a personas con HAS en APS, reduciendo posibles sesgos de medición, así como en la formación uniforme de los investigadores que analizaron los vídeos de las consultas de enfermería. Además, la transparencia en la descripción de los procedimientos y los análisis bivariantes empleados ayudan a evaluar la solidez de los resultados. Estas medidas, en conjunto, refuerzan la calidad metodológica y la credibilidad de las conclusiones del presente estudio.

### Tratamiento y análisis de datos

Inicialmente, se realizó un análisis descriptivo univariante de todas las variables, utilizando medidas de tendencia central (media o mediana) y dispersión (desviación estándar o rango intercuartíl), según corresponda, además de distribuciones de frecuencia para variables categóricas. La normalidad de la distribución de cada variable continua se evaluó formalmente utilizando la prueba de Shapiro-Wilk. Para el análisis bivariado, con el fin de explorar la relación entre el porcentaje de cumplimiento con las etapas del PE y la duración de la consulta, se eligió un enfoque gráfico complementado por un análisis estadístico inferencial. Específicamente, se construyó un diagrama de dispersión con tres elementos:

puntos que representan cada observación, 

líneas de regresión lineal discontinuas para visualizar la tendencia de la relación en cada subgrupo y 

distinción codificada por colores basada en la variable categórica “Número de diagnósticos reportados”. Considerando que la variable ‘duración de la consulta’ violaba la suposición de normalidad (Shapiro-Wilk, p < 0,05), la intensidad y dirección de la asociación continua entre estas dos variables se cuantificaron utilizando el método no paramétrico del coeficiente de correlación de rango (ρ) de Spearman.

Para evaluar la adhesión a las Directrices Brasileñas de Hipertensión Arterial, se realizó un análisis observacional transversal, utilizando el PE como marco metodológico de referencia. Se elaboró una lista de verificación para la extracción de datos que alineaba cada paso del proceso - Evaluación, Diagnóstico, Planificación, Implementación y Evolución de la enfermería - con las recomendaciones específicas de las Directrices. Mediante la observación directa de 29 consultas, se verificó la ejecución de elementos críticos para el manejo de la HAS, como la técnica correcta para medir la presión arterial, la investigación de factores de riesgo cardiovascular, la realización de un examen físico dirigido, la educación sanitaria y el establecimiento de un plan de atención compartida. Así, el PE sirvió como una lente analítica para medir de forma sistemática y exhaustiva si las prácticas observadas correspondían al estándar recomendado de excelencia clínica.

El porcentaje de cumplimiento con las etapas del PE se calculó en función del número de elementos analizados, considerando que todas las etapas tienen el mismo peso. Para ello, se determinó el número total de elementos que componen todo el PE, asegurando una distribución equitativa entre las etapas. Estos porcentajes se describieron y relacionaron gráficamente según las características de los usuarios, los profesionales de enfermería y las características de las consultas. Todos los análisis estadísticos se realizaron utilizando el *software* estadístico R versión 4.1.3 (*Project for Statistical Computing*) junto con el *software* estadístico R Studio versión 1.4.1717 (RStudio).

### Consideraciones éticas

Se cumplieron los preceptos éticos y legales, respetando la Resolución n.º 466/2012 del Consejo Nacional de Salud. El estudio fue aprobado por el Comité de Ética en Investigación (56255622.2.0000.0071) y aprobado por los centros coparticipantes. De acuerdo con *Open Science*, los datos están disponibles mediante consulta parcial con los autores, ya que el registro de las consultas de enfermería puede identificar a las enfermeras, usuarios y unidades sanitarias participantes.

## Resultados

### Usuarios

En el presente estudio, se incluyeron 29 registros de consultas de enfermería relacionados con usuarios de unidades de atención primaria que autorreportaron un diagnóstico de HAS. Los usuarios incluidos en los análisis tenían una edad media de 56,62 años (± 15,28), el 86,21% eran mujeres y el 68,96% eran negros o morenos.

En cuanto a las condiciones socioeconómicas, el 72,41% de los usuarios recibían algún ingreso y, de estos, el 75,87% recibían entre 0 y 2 salarios mínimos. En cuanto a los lugares de origen, tres usuarios eran del municipio situado en el estado de Alagoas (AL), 11 del estado de Amazonas (AM), seis de Rio Grande do Norte (RN) y nueve del estado de São Paulo (SP). Además, el 82,76% declararon vivir con familiares. Aparte de la hipertensión arterial, el 55,27% de los usuarios informaron tener más de tres diagnósticos diferentes. Las principales razones para buscar servicios sanitarios fueron eventos agudos (30,37%), la recogida de pruebas citopatológicas cervicales (17,24%) y la evaluación de los resultados de las pruebas (10,34%).

### Enfermeras

Un total de 13 enfermeras fueron responsables de las consultas con usuarios con HAS: 1 de AL, 4 de AM, 3 de RN y 5 de SP. De estos, el 76,92% eran mujeres. Los servicios de salud en los que trabajaban eran, en el 61,54% de los casos, Unidades Básicas de Salud con una Estrategia de Salud Familiar y, de los 13 profesionales participantes en el estudio, 8 (61,5%) tenían al menos 5 años de experiencia en el área de actividad. Todos los profesionales afirmaron tener algún tipo de posgrado y, de ellos, el 91,54% tenían residencia o especialización en APS, en Salud Familiar y Comunitaria, en Salud Colectiva o en Salud Pública.

En cuanto a facilitar o dificultar los aspectos de las consultas, el 76,92% de las enfermeras tenía una sala exclusiva y el 61,54% tenía acceso a alguna Taxonomía como guía para diagnósticos de enfermería. Otra información sobre las características de las enfermeras cuyas consultas fueron evaluadas en este estudio se muestra en la Tabla 1. Como se ilustra además en esta tabla, hubo casos en los que los enfermeros no informaron o informaron la participación en más de un curso en el último año y la aplicación de múltiples protocolos en la atención a los usuarios.

**Tabla 1:** Perfil profesional del desempeño y la formación de enfermeras que trabajan en los servicios de salud (n = 13). Brasil, 2022-2023

Variables	% (n)*
**Salario** 1 a 2 SM^†^ 3 a 4 SM^†^ 5 a 6 SM^†^ > 7 SM^†^	15,38 (2) 23,08 (3) - 61,54 (8)
**Otra relación laboral** Sí No	38,46 (5) 61,54 (8)
**Tipo de equipo en la Unidad** Estrategia Salud de la Familia UBS^‡^ Mixta UBS^‡^ Tradicional UBS^‡^ Rural	61,54 (8) 15,38 (2) 15,38 (2) 7,69 (1)
**En la Unidad hay un equipo multidisciplinar** Sí No	61,54 (8) 38,46 (5)
**Tiempo de experiencia como enfermero de la APS** ^§^ Entre 1 y 5 años Entre 6 y 10 años > 10 años	3,46 (5) 46,15 (6) 15,38 (2)
**Existen dificultades para llevar a cabo consultas** Sí No	69,23 (9) 30,77 (4)
**Instrumentos estandarizados sobre el Proceso de Enfermería** Sí No	38,46 (5) 61,54 (8)
**Taxonomía utilizada para el Diagnóstico de Enfermería** NANDA^||^ CIPE^¶^ Otros Ninguno	23,08 (3) 30,77 (4) 7,69 (1) 38,46 (5)
**Motivación profesional** Con satisfacción Sin satisfacción	76,92 (10) 23,08 (3)
**Residencia o Especialización en APS** ^§^ Sí No	30,77 (4) 69,23 (9)
**Especialización en Salud de la Familia y Comunidad, Salud Colectiva o Salud Pública** Sí No	61,54 (8) 38,46 (5)
**Número de Profesionales con títulos de maestría** Sí No	30,77 (4) 69,23 (9)
**Participación en cursos en el último año** Salud de los Ancianos Cuidado a pacientes crónicos (HAS**/DM^††^) Proceso de Enfermería	23,08 (3) 30,77 (4) 30,77 (4)
**Tuvo clases sobre el Proceso de Enfermería** Sí No No sé	84,62 (11) 7,69 (1) 7,69 (1)
**En su unidad usan Protocolos de Enfermería** Sí No	46,15 (6) 53,85 (7)
**Protocolos utilizados en la Salud de los Ancianos** Cuaderno de Atención Primaria del Ministerio de Salud Protocolo del Estado Protocolo de otros municipios Protocolo del COREN^‡‡^ Protocolo da su secretaría municipal de salud	76,92 (10) 7,69 (1) 7,69 (1) - 15,38 (2)
**Protocolos utilizados para atención a Crónicos** Cuaderno de Atención Primaria del Ministerio de Salud Protocolo Ministerio de Salud Protocolo del Estado Protocolo de otros municipios Protocolo del COREN^‡‡^ Protocolo de su Secretaría Municipal de Salud	76,92 (10) 61,54 (8) 23,08 (3) 7,69 (1) - 15,38 (2)

*%(n) = Valor porcentual (número bruto); ^†^SM = Salario mínimo, correspondiente a R$1518,00 en Brasil, año 2025; ^‡^UBS = Unidad Básica de Salud; ^§^APS = Atención Primaria en Salud; ^||^NANDA = *North American Nursing Diagnosis Association*; ^¶^CIPE = Clasificación Internacional para la Práctica de Enfermería; **HAS = Hipertensión Arterial Sistémica; ^††^DM = Diabetes Mellitus; ^‡‡^COREN = Consejo Regional de Enfermería

El tiempo medio de cada consulta fue de 22,89 minutos (± 13,54), el 66,67% de ellos se interrumpieron durante la consulta, el 55,17% estaban programados previamente y la queja del usuario estaba relacionada con HAS en el 24,14% de las atenciones.

La siguiente descripción trata sobre el contenido generado a partir del análisis de los vídeos de las consultas, según la lista de comprobación. En la etapa de Evaluación de Enfermería, el 72,41% de los profesionales no midió su presión arterial durante el tratamiento y, de los que lo hicieron, el 25% realizó este procedimiento con la técnica correcta. La búsqueda de datos antropométricos estuvo ausente en el 51,72% de las consultas, así como en la auscultación cardíaca (86,21%). La evaluación de las extremidades de los usuarios estuvo presente en el 10% de las consultas, en las que los profesionales investigaron la posibilidad de la presencia de edema.

Los profesionales realizaron la evaluación de exámenes complementarios básicos en el 31,0% de las consultas y en el 88,89% de ellas el resultado se comunicó al usuario. El uso de medicamentos continuos se investigó en el 51,72% de las consultas y en ninguna de ellas se cuestionó el uso de medicamentos de venta libre y/o el uso de drogas ilícitas.

Se exploraron factores de riesgo específicos para enfermedades cardiovasculares reales en el 72,42% de las consultas, siendo la edad la más frecuente (66,67%). La presencia de tabaquismo, consumo de alcohol o consumo cuantitativo de sodio no se exploró en el 94,24%, 95,25% y 95,24% de las consultas, respectivamente. La enfermedad crónica de las arterias coronarias y renales estuvieron presentes en 1 y 3 visitas, respectivamente. La investigación de la presencia de diabetes se evidenció en el 42,86% de las consultas.

En el 79,31% de las consultas, no se investigaron los aspectos psicosociales, culturales y socioeconómicos que podrían asociarse con los valores y comorbilidades de la medición de la presión arterial y de los que sí incluyeron dicha investigación, el estrés emocional fue el más frecuente (66,67%).

Estuvieron ausentes en el 100% de las consultas: evaluación del Índice Tobillo-Brazo (ITB), auscultación de las arterias carótidas, cálculo de la Tasa de Filtración Glomerular (TFG), examen neurológico, antecedentes de Accidente Cerebrovascular (ACV) e investigación de retinopatía hipertensiva, cálculo de la Velocidad de Onda de Pulso (VOP), evaluación del choque de la punta (*ictus cordis*) a la palpación, investigación de antecedentes familiares de inicio temprano de HTA, investigación de apnea del sueño y de presión de pulso.

La comunicación del Diagnóstico de Enfermería no fue evidente en ninguna de las consultas. En cuanto a la fase de planificación, el 89,66% de los profesionales no establecieron objetivos y la planificación se elaboró junto con el usuario en el 20,69% de las atenciones.

La Prescripción de Enfermería no se verbalizó en el 75,86% de las consultas y en ninguna de ellas se entregó por escrito al usuario. Finalmente, el cálculo del riesgo cardiovascular no fue evidente en ninguna de las consultas. El 65,62 % de las consultas fueron de seguimiento y la etapa de Evolución en Enfermería no fue explícita en ninguna de ellas.

En cuanto a las habilidades de comunicación y relaciones interpersonales de los profesionales, el 65,52% identificó al usuario y el 86,21% no se presentó. La comodidad y la privacidad estuvieron garantizadas en el 96,55% de las consultas y la entrevista comenzó con preguntas abiertas en el 51,72% de las consultas. Además, el 79,31% de los profesionales mantuvo contacto visual con el usuario durante la interacción y ofreció tiempo suficiente para que el usuario se expresara, sin interrupciones.

Además, el 58,62% de los profesionales realizaron escucha activa y el 93,1% utilizaron un lenguaje adaptado a las condiciones educativas y socioeconómicas de los usuarios. La verificación de la comprensión de la persona y el estímulo para aclarar dudas estuvieron presentes en el 79,31% de las consultas y la técnica de sumarización se aplicó en el 55,17% de las reuniones clínicas. Aun, el 75,86% de los usuarios no participaron en la planificación de opciones terapéuticas relacionadas con el propio proceso de atención a la enfermedad y no se solicitó retroalimentación al usuario sobre la atención proporcionada en el 89,66% de las consultas.

### Proceso de Enfermería

*Según la ejecución*

La Tabla 2 muestra que, en resumen, de un total de 48 ítems evaluados por la lista de verificación, se encontró que la mediana de los ítems cubiertos era 18, lo que indica que el 50% de las enfermeras cumplieron con hasta 18 ítems del Proceso de Enfermería. Este valor corresponde a una mediana de cumplimiento del 20% en relación con el número total de etapas del PE.

**Tabla 2:** Distribución del cumplimiento en las etapas del Proceso de Enfermería, según los ítems propuestos por las Directrices Brasileñas sobre Hipertensión Arterial (N = 29). Brasil, 2022-2023

Variables	Sí % (n)*	No % (n)*
**Evaluación de enfermería realizada por los profesionales**		
¿Le ha tomado la tensión arterial durante la consulta?	27,59 (8)	72,41 (21)
¿Buscaste datos antropométricos del usuario?	48,28 (14)	51,72 (15)
¿Has tenido auscultación de la arteria carótida?	-	100,0 (29)
¿Realizaste auscultación cardíaca?	13,79 (4)	86,21 (25)
¿Has contado la frecuencia cardíaca?	3,45 (1)	96,55 (28)
¿Has examinado las extremidades?	10,34 (3)	89,66 (26)
¿Has realizado una evaluación de ITB^†^?	-	100,0 (29)
¿Has comprobado los resultados de pruebas complementarias básicas relacionadas con la hipertensión o la RCV^‡^?	31,03 (9)	68,97 (20)
¿Evaluó la función renal mediante el cálculo de la TFG^§^ con CKD-EPI^||^ o MDRD^¶^?	-	100,0 (29)
¿Te has examinado el abdomen?	6,9 (2)	93,1 (27)
¿Te han hecho un examen neurológico?	-	100,0 (29)
¿Has investigado medicamentos en uso continuo?	51,72 (15)	48,28 (14)
¿Has investigado el uso de medicamentos sin prescripción médica?	-	100,0 (29)
¿Investigó el consumo de drogas ilícitas?	-	100,0 (29)
¿Investigaron factores de riesgo específicos para enfermedades cardiovasculares reales?	72,41 (21)	27,59 (8)
¿Has investigado factores que puedan modificar el riesgo de usuarios hipertensos?	27,59 (8)	72,41 (21)
¿Investigaron aspectos psicosociales, culturales y socioeconómicos que puedan estar interfiriendo con los valores/comorbilidades medidos?	20,69 (6)	79,31 (23)
¿Has realizado una evaluación de VOP**?	-	100,0 (29)
¿Has evaluado *ictus cordis* en la palpación?	-	100,0 (29)
**Diagnóstico de Enfermería**		
Listó diagnósticos de enfermería:	-	100,0 (29)
¿Comunicaste el(los) diagnóstico(s) listados al usuario?	-	100,0 (29)
**Planificación de la enfermería**		
¿Habéis determinado resultados que se espera alcanzar y/o fijado metas?	10,34 (3)	89,66 (26)
¿Has establecido acciones o intervenciones de enfermería que se llevarán a cabo para alcanzar los objetivos?	33,33 (1)	66,67 (2)
**Implementación de la atención de enfermería**		
¿Necesita el usuario solicitar exámenes básicos complementarios?	17,24 (5)	82,76 (24)
¿Realizó la prescripción de enfermería? (Ya sea por escrito o verbal)	24,14 (7)	75,86 (22)
De los que realizaron la prescripción (n=7), ¿se entregó al usuario por escrito en papel?	-	100,0 (7)
**Evolución de enfermería**		
¿Evaluó la atención de enfermería?	-	100,0 (29)
¿Has listado nuevos diagnósticos de enfermería para la necesidad que se está presentando?	-	100,0 (29)

*%(n) = Valor porcentual (número bruto); ^†^ITB = Índice Tobillo-Braquial; ^‡^RCV = Riesgo Cardiovascular; ^§^TFG = Tasa de filtración glomerular; ^||^CKD-EPI = Colaboración de Epidemiología de Enfermedades Renales Crónicas (*Chronic Kidney Disease Epidemiology Collaboration*), ecuación para estimar la tasa de filtración glomerular; ^¶^MDRD = Modificación de la dieta en la enfermedad renal (*Modification of Diet in Renal Disease*), fórmula para estimar la tasa de filtración glomerular; **VOP = Velocidad de onda de pulso

Como se ilustra en la Figura 2, se observó una correlación positiva y estadísticamente significativa entre una mayor duración de la consulta de enfermería y una mayor adherencia a las etapas del Proceso de Enfermería (PE) en pacientes con HAS. El análisis del tiempo de consulta utilizando la prueba de Shapiro-Wilk confirmó la ausencia de normalidad en su distribución (p = 0,0121). En vista de este resultado, optamos por utilizar la prueba no paramétrica de Spearman para evaluar la correlación.

**Figura 2:** Correlación entre la duración de la consulta de enfermería y el cumplimiento de las etapas del Proceso de Enfermería con usuarios con hipertensión arterial en la Atención Primaria de Salud. Brasil, 2022-2023

El análisis estratificado por complejidad diagnóstica (Figura 3) reveló un gradiente de magnitud de correlación, aunque sin significación estadística en ninguno de los subgrupos (p > 0,05). Específicamente, se observó una correlación positiva moderada (ρ = 0,521; p = 0,150) en el grupo con solo HAS, una correlación positiva muy débil (ρ = 0,138; p = 0,653) en el grupo con diagnóstico adicional, y una correlación positiva de fuerte magnitud (ρ = 0,685; p = 0,090) en el grupo con mayor complejidad (2-3 diagnósticos adicionales). Este patrón ascendente sugiere que la relación entre el tiempo de consulta y la adhesión a la enfermera practicante se intensifica progresivamente con el aumento de la complejidad del cuadro clínico.

**Figura 3:** Correlación entre los diagnósticos asociados a hipertensión arterial y el tiempo de consulta necesario para cumplir con las etapas del Proceso de Enfermería. Brasil, 2022-2023

#### Según el registro

Según las etapas del PE, el 79,31% de las consultas se describieron en los historiales médicos y el contenido era legible en el 87,5% de los registros. En la Evaluación, los datos subjetivos estuvieron ausentes en el 52,17% de las consultas y los datos objetivos en el 86,96% de ellas. El 73,91% de las consultas no tenían registro del diagnóstico de enfermería y el 95,65% de ellas contenían al menos un diagnóstico según la Clasificación Internacional de Atención Primaria (CIAP).

En cuanto a la fase de planificación, en el 95,65% de las consultas no hubo registro de objetivos y, de los que sí, uno de ellos estaba relacionado con el diagnóstico de enfermería mencionado anteriormente. La prescripción de enfermería estaba presente en dos registros de consultas, que eran coherentes con los objetivos y diagnósticos de enfermería previamente establecidos. La etapa de Implementación estuvo ausente en el 95,65% de los registros, y la etapa de Evolución de la Enfermería fue evidente en el 50% de ellos.

## Discusión

Los resultados de este estudio indican una brecha significativa entre la práctica observada en la Atención Primaria de Salud y los estándares de atención recomendados por las Directrices Brasileñas de Hipertensión Arterial, cuando se analizan a través del prisma del Proceso de Enfermería (PE). La baja frecuencia de realizar componentes esenciales de la evaluación -como la medición técnica de la presión arterial, la investigación de factores de riesgo modificables (como la ingesta de sodio y el estilo de vida sedentario) y el examen físico dirigido- sugiere que la atención se realiza de manera mecanizada y fragmentada, sin aprovechar la consulta como una oportunidad para capturar de forma completa y precisa el perfil de riesgo cardiovascular del usuario.

La casi ausencia de la formulación de diagnósticos de enfermería (100% no listados) es especialmente preocupante, ya que demuestra que el juicio clínico no se está formalizando, impidiendo que la planificación del cuidado sea específica, individualizada y basada en las necesidades reales de salud identificadas. Tal fragilidad se reveló desde el momento de la Evaluación de Enfermería, que ocurre de forma incompleta. En consecuencia, considerando que las etapas del PE están interrelacionadas e interdependientes, la realización de las etapas posteriores se ve comprometida. Este resultado es coherente con los hallazgos de un estudio que realizó un diagnóstico situacional del rendimiento de las enfermeras en la APS en Santa Catarina^18^ realizado en 2023, que concluyó que la aplicación de la enfermería en las consultas de enfermería aún es incipiente y fragmentada.

La desconexión entre la recogida de datos y los pasos posteriores del PE resulta en un ciclo de atención incompleto. La ausencia de diagnósticos clínicos sistematizados compromete la planificación, que suele ser inespecífica o ausente, como lo demuestra la baja tasa de establecimiento de objetivos conjuntos (10,34%). Este escenario ya se ha descrito en otros contextos, en los que la elaboración de planes de atención individualizados resultó ser un diferencial en la práctica de la atención, favoreciendo la adhesión y el control de la hipertensión^19^ ^-^ ^20^. En consecuencia, las intervenciones de enfermería implementadas carecen de una base teórica y una dirección clara, centrándose en las solicitudes de pruebas y prescripciones genéricas, en detrimento de las estrategias educativas y conductuales. Sin embargo, la evidencia sólida indica que las intervenciones educativas dirigidas por enfermeros reducen significativamente los niveles de presión arterial y favorecen el autocuidado^8^ ^,^ ^21^ ^-^ ^22^.

La alta proporción de profesionales que no proporcionaron retroalimentación o no verificaron la comprensión del usuario (89,66%) refuerza la comunicación unidireccional, que no promueve la corresponsabilidad. Los estudios cualitativos también muestran que, en la práctica, la Consulta de Enfermería tiende a ser poco creativa y centrada en lo profesional, limitando la participación activa del usuario^23^. Así, se observa que la brecha en la integración entre diagnóstico, planificación e intervención tiene un impacto directo en la eficacia de la atención en condiciones crónicas, reforzando la necesidad de reorganizar el proceso de trabajo para garantizar una atención centrada en el usuario y basada en la evidencia.

Con el fin de analizar los factores relacionados con la aplicación completa del PE, se consideraron las características de los usuarios atendidos, las enfermeras y las consultas evaluadas. Un aspecto importante fue el tiempo de consulta, ya que era evidente que las personas con múltiples comorbilidades necesitan un tiempo de consulta más largo para que todas las necesidades de salud estén cubiertas. Además, hubo una interrupción en la mayoría de las consultas, lo que desorganiza el razonamiento clínico y perjudica la gestión del tiempo, como lo demuestra una revisión integrativa de la literatura realizada en 2020^24^.

Así, se introduce en el contexto de la comunicación clínica, un punto importante en las consultas de enfermería, pero que en este estudio se evalúa como algo secundario. Acciones como comunicar diagnósticos y objetivos terapéuticos, entregar la prescripción escrita de enfermería y evaluar la atención con el usuario son esenciales para una comunicación clínica eficaz^25^ ^-^ ^26^, para la adherencia al tratamiento^27^ y para mejorar el vínculo y la satisfacción^28^. Sin embargo, gestionar la agenda y gestionar las interferencias externas que acaban siendo responsabilidad de las enfermeras en la APS sigue siendo un reto para la plena implementación de la enfermería especializada y para la ejecución de la consulta de enfermería, que incluye todos los puntos recomendados por las Directrices de la HAS^29^.

Un análisis de consultas de enfermería realizado en Río de Janeiro en 2021 evidenció que los profesionales estaban sobrecargados por la presión de los cuidados y agendas ocupadas, lo que resultó en un modelo de atención centrada en la medicina enfocado principalmente en la conducta de la queja^30^, lo que dificulta el uso completo del PE y puede interferir negativamente en los resultados clínicos de los usuarios.

 Se destaca el incumplimiento de la etapa de Diagnóstico de Enfermería. En nuestro estudio, esta etapa estuvo presente en el 26,09% de las consultas evaluadas y justifica este hecho con la falta de habilidad y gestión en las taxonomías clasificatorias. Sin embargo, el 38,5% de los profesionales evaluados informaron no utilizar ninguna taxonomía para el diagnóstico de enfermería. Esta evidencia dialoga con los resultados en otros contextos, donde la dificultad para implementar la etapa diagnóstica en el PE sigue estando marcada por la complejidad teórica, lagunas en la formación, falta de apoyo institucional, alta carga de trabajo y bajo dominio práctico^31^ ^-^ ^32^.

Aunque la mayoría de los profesionales informan haber recibido formación de grado en Proceso de Enfermería y tener especialización y/o residencia, se ha verificado que existe una distancia entre la teoría y el uso real de esta herramienta por parte de por parte de ellos, siendo el principal obstáculo la falta de formación y la aplicación del conocimiento en la práctica^33^, entendiendo que el PE es una herramienta para la práctica segura de las enfermeras. Por lo tanto, es necesario conocer las Taxonomías que estandarizan la atención de enfermería específica, así como los estándares de atención en los Programas de Salud, porque los protocolos generales, por ejemplo, no garantizan la apropiación completa del PE en la realización del encuentro clínico durante la consulta.

Así, aunque muchos profesionales participantes en el estudio informaron que utilizaban algún protocolo para guiar la atención, más de la mitad no eran específicos de la enfermería, lo que puede ser un obstáculo para la consolidación de un PE cualificado^34^. El uso de protocolos de enfermería es fundamental, ya que ayudan en la toma de decisiones con un enfoque en la seguridad del paciente y apoyan al profesional, teniendo en cuenta los principios éticos de la profesión^35^. La necesidad de implementar protocolos como estrategias para reducir el número de obstáculos y fortalecer el proceso^36^ sería de gran importancia, ya que el 53,85% de las enfermeras informaron no haber utilizado los Protocolos de Enfermería durante su práctica de cuidados.

En grandes municipios del interior del estado de São Paulo^37^, en 2015, el PE en una unidad de APS aún se implementaba de forma incipiente, con un 19% de enfermeros que declaraban no usarlo, un 38% evaluando que lo usaban raramente y un 29% a veces. En comparación con nuestros resultados, hubo una mejora en el uso del PE, aunque no incluye sus cinco etapas. Por tanto, es esencial que, en relación con la elaboración de directrices y protocolos clínicos, se calcule y evidencie el tiempo necesario para cumplir con las actividades planificadas, ya que esto podría incluso implicar el grado de recomendación de una determinada intervención, la priorización de casos más graves y la necesidad de aumentar el número de profesionales disponibles para atender a estos usuarios^38^.

A pesar de los desafíos para el PE en la APS durante el cuidado de la persona con HAS, las consultas de enfermería son prácticas asistenciales que favorecen la atención longitudinal, ya que las intervenciones se basan en evidencia científica y se dirigen a aspectos clínicos, sociales, culturales y regionales de la atención, de manera personalizada^39^ ^-^ ^41^.

A partir del PE, basado en teorías y utilizada en su totalidad, desarrollado junto con la persona, a nivel individual, hay una mayor satisfacción con el seguimiento de la salud, lo que favorece cambios de comportamiento y mejora de la situación clínica y la calidad de vida^42^ ^-^ ^43^. Para los profesionales, la consulta de enfermería, a pesar de los desafíos, aumenta la autonomía de la enfermera y produce experiencias laborales positivas^44^ ^-^ ^45^. En el ámbito de las políticas públicas y del Sistema Único de Salud (SUS, por sus siglas en portugués), la APS puede fortalecerse aún más, con una contribución significativa de la enfermería para reducir la carga de enfermedades crónicas no transmisibles. 

Este estudio tiene algunas limitaciones. Primero, no se calculó el tamaño de la muestra y la selección de las unidades se realizó por conveniencia. Sin embargo, se incluyeron unidades de 4 municipios diferentes del país que presentan características sociodemográficas particulares de las regiones de Brasil. La segunda limitación se refiere al número de consultas analizadas (n=29) y al número de enfermeras (n=13), lo que resulta en características heterogéneas de los profesionales y un espectro limitado. El posible sesgo informativo relacionado con el instrumento utilizado para evaluar las consultas de enfermería puede ser una limitación.

Finalmente, como en los estudios observacionales, existe la posibilidad de un sesgo de selección atribuible a las enfermeras que aceptaron participar, que pueden presentar prácticas diferentes en relación con quienes se negaron a participar en la investigación. Además, existe la posibilidad de un sesgo de deseabilidad social, que puede desencadenarse por cambios de comportamiento al participar en la investigación y estar sujeto a registro y observación. A pesar de las limitaciones, el presente estudio analiza resultados importantes respecto a la evaluación de todas las etapas del PE en la APS en el cuidado de personas con HAS. Los hallazgos contribuyen a la reflexión crítica sobre la atención de enfermería, además de señalar la importancia de registrar el PE. Presenta los factores asociados a los desafíos de utilizar el PE, que guiarán las acciones y la investigación y permitirán la adaptación de documentos y políticas públicas para contemplar las especificidades de la enfermería en la APS.

Debido al aún escaso número de estudios realizados en Brasil sobre este tema, esta investigación, como estudio principal, abre caminos e ilumina futuras discusiones para la producción de instrumentos y conocimientos que refuercen la práctica clínica de enfermería. De este modo, su objetivo es impactar positivamente en la razón de existir como categoría profesional: proporcionar la mejor atención a los usuarios, basada en instrumentos sólidos y en la mejor evidencia científica.

## Conclusión

Se evidenció debilidad en la evaluación de los elementos recomendados por las Directrices Brasileñas de Hipertensión Arterial y en la consolidación del PE durante las consultas de enfermería con personas con HAS. La complejidad de los usuarios, el tiempo y la interrupción de las consultas, la alta demanda de atención, la formación de profesionales y la falta de protocolos estructurados de enfermería basados en las etapas del PE fueron hipótesis planteadas para justificar este hallazgo. Además, la publicación de guías de cuidado sin un documento guía para su aplicabilidad es un obstáculo para su aplicación real en la vida diaria.

## Data Availability

Todos los datos generados o analizados durante este estudio están incluidos en este artículo publicado.
